# Wireless Stimulation of Barium Titanate@PEDOT Nanoparticles Toward Bioelectrical Modulation in Cancer

**DOI:** 10.1021/acsami.4c12387

**Published:** 2025-01-29

**Authors:** Catarina Franco Jones, Marta S. Carvalho, Akhil Jain, Paula Rodriguez-Lejarraga, Filipa Pires, Jorge Morgado, Senentxu Lanceros-Mendez, Frederico Castelo Ferreira, Teresa Esteves, Paola Sanjuan-Alberte

**Affiliations:** † Department of Bioengineering and iBB - Institute of Bioengineering and Biosciences, Instituto Superior Técnico, Universidade de Lisboa, Av. Rovisco Pais, Lisbon 1049-001, Portugal; ‡ Associate Laboratory i4HB−Institute for Health and Bioeconomy, Instituto Superior Técnico, Universidade de Lisboa, Av. Rovisco Pais, Lisbon 1049-001, Portugal; § Division of Pharmacy and Optometry, Faculty of Biology, Medicine and Health, University of Manchester, Oxford Road, Manchester M13 9PT, U.K.; ∥ Basque Center for Materials, Applications and Nanostructures, UPV/EHU Science Park, BCMaterials, Leioa 48940, Spain; ⊥ Department of Bioengineering and Instituto de Telecomunicações (IT), Instituto Superior Técnico, Universidade de Lisboa, Lisboa 1049-001, Portugal; # Centre of Physics Universities of Minho and Porto (CFUM-UP), University of Minho and Laboratory of Physics for Materials and Emergent Technologies, LapMET, Campus de Gualtar, Braga 4710-057, Portugal; ∇ Ikerbasque, Basque Foundation for Science, Bilbao 48009, Spain

**Keywords:** cancer bioelectricity, nanobioelectronics, multifunctional nanoparticles, wireless stimulation, breast cancer, ultrasound stimulation

## Abstract

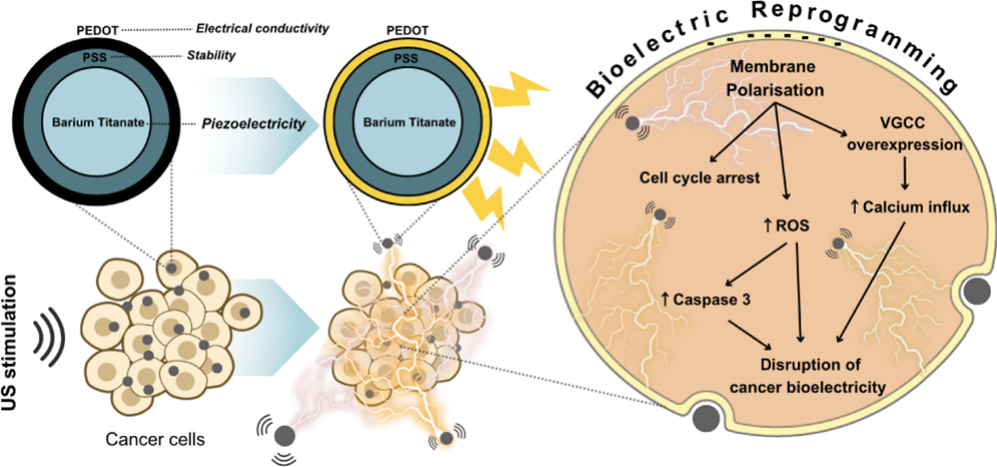

Cancer cells possess distinct bioelectrical properties, yet therapies leveraging these characteristics remain underexplored. Herein, we introduce an innovative nanobioelectronic system combining a piezoelectric barium titanate nanoparticle core with a conducting poly(3,4-ethylenedioxythiophene) shell (BTO@PEDOT NPs), designed to modulate cancer cell bioelectricity through noninvasive, wireless stimulation. Our hypothesis is that acting as nanoantennas, BTO@PEDOT NPs convert mechanical inputs provided by ultrasound (US) into electrical signals, capable of interfering with the bioelectronic circuitry of two human breast cancer cell lines, MCF-7 and MDA-MB-231. Upon US stimulation, the viability of MCF-7 and MDA-MB-231 cells treated with 200 μg mL^–1^ BTO@PEDOT NPs and US reduced significantly to 31% and 24%, respectively, while healthy human mammary fibroblasts (HMF) were unaffected by the treatment. Subsequent assays shed light on how this approach could interact with cell’s bioelectrical mechanisms, namely, by increasing intracellular reactive oxygen species (ROS) and calcium concentrations. Furthermore, this system was able to polarize cancer cell membranes, halting their cell cycle and potentially harnessing their tumorigenic characteristics. These findings underscore the crucial role of bioelectricity in cancer progression and highlight the potential of nanobioelectronic systems as an emerging and promising strategy for cancer intervention.

## 1 Introduction

Bioelectrical properties of cancer cells have emerged as a promising target for novel therapeutic strategies.^1,2^ Cancer cells exhibit an aberrant bioelectrical behavior with a key role in its pathophysiology, characterized by a more depolarized membrane linked to their growth and proliferation.^3−5^ This has been associated with the overexpression of ionic channels^2^ including calcium,^6^ sodium,^7^ potassium,^8^ chloride,^9^ and piezo channels;^10^ and dysregulation of faradaic currents that stem from a heightened metabolic activity.^2,11,12^ As a result, it has been shown that in breast cancer biopsy samples, the resting membrane potential is approximately −13 mV, markedly depolarized in comparison to the approximately −60 mV observed in he breast cells.^13^

By modulating the bioelectrical state of cancer cells using bioelectronics, it is possible to regulate their growth and proliferation.^14^ For instance, Tumor Treating Fields (TTFs) use high-frequency alternating currents to disrupt cell division.^15^ This technology is used for the treatment of glioblastoma type IV, a very lethal brain cancer, and has been shown to increase life expectancy in conjunction with conventional therapies.^16^ However, its use presents limitations and side effects and is not yet translatable to other tumors.^16,17^ Wireless nanobioelectronics, instead, present greater spatial resolution without requiring physical connection to power supplies.^2^ Therefore, they have the potential to target cancer bioelectricity in a noninvasive manner. This nanoscale precision allows for more direct interaction with cellular processes.^2,18^ However, the development of such systems is in its infancy.

Building upon such innovations, our research introduces a novel wireless nanobioelectronic system based on piezoelectric barium titanate nanoparticles (BTO) as a building block. BTO has already demonstrated its potential to be remotely activated under external ultrasounds (US)^19^ and its antitumorigenic capabilities have shown promising results across various cancer models.^20−24^ Additionally, nanoparticles (NPs) have the advantage that they could bypass drug efflux mechanisms of cancer cells^25^ due to their small size and surface modification capabilities, increasing their retention within cells, or even achieve controlled and sustained release of trapped anticancer agents.^26^ The mechanism of cellular uptake of BTO, typically involves endocytosis.^27^ Studies have shown that naked BTO may display low cellular uptake efficiency^28^ and modifications such as coating with polymers can enhance this.^29^

Previously, Wang et al. designed NPs consisting of a BTO core surrounded by a poly(3,4-ethylenedioxythiophene) (PEDOT) shell (BTO@PEDOT NPs), with the aim to develop a material with a high dielectric constant and low dielectric loss for applications in miniaturized electronics.^30^ In fact, BTO@PEDOT NPs have immense potential in bioelectronics, due the high biocompatibility of these materials;^31,32^ yet their biological applications have never been explored to harness their therapeutic potential.

In this work, we present a wireless nanobioelectronic system consisting of BTO@PEDOT NPs remotely activated by external US with the ability to disrupt cancer bioelectricity. US are widely employed in clinical settings, evolving from their traditional diagnostic applications to alternative therapies. In oncology, High-Intensity Focused Ultrasound (HIFU) is the most common US modality.^33^ HIFU delivers targeted thermal energy for tumor ablation and necrosis. In contrast, our approach utilizes Low-Intensity Pulsed Ultrasound (LIPUS), which operates at much lower intensities and is primarily applied in the tissue regeneration, drug delivery, and fracture healing fields, with less direct application in cancer therapy.^34−36^ Herein, we repurpose the US not as a direct therapeutic agent—given that the frequency and intensity utilized are lower than those typically employed in therapeutic applications—but as a noninvasive approach to activate our NP system. Furthermore, unlike sonodynamic therapy (SDT), which relies on sonosensitizers to generate reactive oxygen species (ROS) upon US activation, our approach leverages the intrinsic piezoelectric properties of BTO@PEDOT NPs to interfere directly with cancer cell bioelectricity.

To achieve this, core–shell BTO@PEDOT NPs were initially synthesized. The choice of PEDOT as shell material is critical^37^ for enhancing the overall efficacy of the BTO,^38^ as it provides excellent chemical and electrochemical stability^39^ and biocompatibility,^40^ and it amplifies the electrical response of the BTO@PEDOT NPs under US stimulation by homogenizing the electrical potential developed by the piezoelectric core under US.^41,42^ Next, the anticancer potential of the BTO@PEDOT NPs under US was evaluated on two breast cancer cell models, MCF-7 and MDA-MB-231, and a healthy model, primary human mammary fibroblasts (HMF). Finally, the mechanisms underlying the cell responses, including ROS production, caspase-3 activity, membrane polarization, intracellular calcium concentrations and cell cycle dynamics were evaluated to establish links with cancer bioelectricity, confirming the disruption of the bioelectrical signals of cancer cells induced by the US-stimulated BTO@PEDOT NPs.

## 2 Materials and Methods

### 2.1 Materials

BaTiO_3_ nanoparticles (BTO, 99.9%, Tetragonal), with an average diameter of 200 and 500 nm were supplied by US Research Nanomaterials, Inc., USA. α-bromoisobutyryl bromide (BiBB), aminopropyl trimethoxysilane (γ-APS), 2,2′-bipyridine, sodium 4-styrenesulfonate (SS), *N*-hydroxysuccinimid, copper(I) bromide, 3,4-ethylenedioxythiophene (EDOT), iron(III) chloride (FeCl_3_),hydrogen peroxide (35% H_2_O_2_), WST-8 cell counting kit and the Fluo-4, AM, cell permeant were purchased from Sigma-Aldrich. Fetal bovine serum (FBS), l-glutamine, penicillin-streptomycin solution (5.000 U mL^–1^), Roswell Park Memorial Institute (RPMI) 1640 Medium, Dulbecco’s Modified Eagle Medium/Nutrient Mixture F-12 (DMEM F-12), LIVE/DEAD Viability/Cytotoxicity Kit, CellsDirect One-Step qRT-PCR Kit, High-Capacity cDNA Reverse Transcription kit and DiBAC_4_(3) (bis(1,3-dibutylbarbituric acid)trimethine oxonol) were purchased from ThermoFisher.

DCFDA/H2DCFDA—Cellular ROS Assay Kit (ab113851), Cell Cycle Analysis Kit (ab287852) and Caspase-3 Assay Kit (Colorimetric) (ab39401) were purchased from Abcam. RNeasy Mini Kit was purchased from QIAGEN. MCF-7 cells were obtained from the Stem Cell Engineering and Regenerative Medicine Research Group (SCERG) cell bank of the Instituto Superior Técnico (Lisbon). MDA-MB-231 cells were purchased from ATCC and primary human mammary fibroblasts (HMF) were purchased from Innoprot (Spain, ref # P10893).

### 2.2 BTO@PEDOT NPs Synthesis

The synthesis of the nanobioelectronic system consisting of a BTO core surrounded by a PEDOT shell (BTO@PEDOT NPs) was adapted from a previously described protocol^30^ consisting of four main steps: hydroxylation of BTO (BTO–OH), bromination (BTO-Br), surface-initiated atom transfer radical polymerization (SI-ATRP) of polystyrenesulfonate (PSS, BTO-PSS), and finally the chemical oxidative polymerization of EDOT (BTO@PEDOT NPs).

Briefly, a solution of 0.179 g mL^–1^ of either 200 or 500 nm BTO in H_2_O_2_ was prepared in a round-bottom flask. The mixture was sonicated for 30 min and refluxed at 105 °C overnight. The particles were recovered through centrifugation at 10000 rpm for 8 min and washed three times with deionized water. BTO–OH NPs were dried under vacuum at 80 °C overnight. A suspension of 0.179 g mL^–1^ of BTO–OH NPs in toluene was subsequently prepared in a round-bottom flask. The mixture was sonicated for 30 min and 7.9% (v/v) of γ-APS was added. The mixture was heated to 80 °C for 24 h under N_2_ atmosphere. The particles were recovered through centrifugation at 10000 rpm for 8 min and washed three times with toluene and dried under vacuum at 80 °C overnight. A suspension of the previous BTO–OH NPs in CH_2_Cl_2_ (0.2 g mL^–1^) dichloromethane was prepared and sonicated for 1 h, and 1.45% (v/v) of triethylamine (Et_3_N) was added to the previous suspension at 0 °C. In parallel, a 10.08% (v/v) of BiBB in CH_2_Cl_2_ was prepared. This mixture was then added dropwise to the previous dispersion at 0 °C and stirred at 0 °C for 3 h and then at room temperature for the following 20 h. The BTO-Br NPs were recovered through centrifugation at 10000 rpm for 8 min and washed three times with CH_2_Cl_2_ and dried under vacuum at 80 °C overnight. A 0.045 mg mL^–1^ dispersion of BTO-Br NPs was prepared in a solvent mixture of water:methanol (3:1, v/v), under sonication for 30 min in a Schlenk. Several ratios of SS to BTO-Br NPs were added to the mixture, as shown in Table S1, as well as 6.16 mg mL^–1^ of 2,2′-bipyridine. The mixture was degassed with a nitrogen purge for 30 min with continuous stirring, followed by the addition of 2.88 mg mL^–1^ of CuBr under N_2_ atmosphere. The reactant mixture was degassed by three freeze-thaw cycles, backfilled with nitrogen and stirred at 25 °C overnight. The BTO-PSS NPs were recovered through centrifugation at 10000 rpm for 8 min and washed three times with deionized water and dried under vacuum at 80 °C for 3 days. A dispersion of 0.04 g mL^–1^ of BTO-PSS NPs in deionized water was prepared in an ultrasonic bath for 30 min. According to the ratio studied (Table S1), different amounts of EDOT and FeCl_3_ were added to the previous mixture. The feeding ratio of EDOT: SS was always kept at 2:1. The mixture was stirred for 41 h at room temperature. Following this, acetone was added to the mixture in a 3:1 ratio. The BTO@PEDOT NPs were collected by filtration with a Buchner funnel and the mixture was washed with ethanol:water (1:2, v/v) until the filtrate was transparent. The final product was washed with deionized water. The dry filter cake was dried under vacuum at room temperature for 24 h. A black powder of BTO@PEDOT NPs was obtained after manual grinding.

### 2.3 BTO@PEDOT NPs Characterization

The BTO@PEDOT NPs were analyzed through dynamic light scattering (DLS, Nano ZS90, Malvern Instruments, Worcester, UK) at 25 °C in Milli-Q water using a He–Ne laser of 633 nm and a detector angle of 173°. Each sample was prepared at a concentration of 1 mg mL^–1^ in miliQ water and sonicated prior to the analysis.

The morphology of the BTO@PEDOT NPs was analyzed by transmission electron microscopy (TEM). Samples at 1 mg mL^–1^ were sonicated for 15 min for dispersion in water, and 10 μL were placed on a carbon film coated 200 mesh copper grid (EM resolution) for 1 h, followed by placing another 5 μL of sample after 30 min. The measurements were performed in a JEOL 2100+ TEM equipped with an Oxford Instruments X-MaxN 80 TLE EDS detector for elemental analysis, operating at 200 kV accelerating voltage. JEOL 2100+ TEM is also equipped with bright field STEM detector and a HADDF (high-angle annular dark-field scanning-TEM) detector.

Additionally, BTO@PEDOT NPs were analyzed by ATR–FTIR using a Spectrum Two FT-IR Spectrometer (PerkinElmer, Waltham, MA, USA), equipped with a Pike Technologies MIRacle ATR accessory (Fitchburg, WI, USA). Transmittance spectra were obtained from 400 to 4000 cm^–1^ (resolution of 4 cm^–1^ accumulation of 8 scans) at room temperature and an automatic baseline correction treatment was applied using the acquisition software.

### 2.4 Piezoresponse Force Microscopy

200 and 500 nm BTO and BTO@PEDOT NPs underwent dispersion in water utilizing an ultrasonic bath. Subsequently, a droplet of this dispersion was deposited onto an Indium–Tin Oxide (ITO) coated glass substrate. Piezoresponse Force Microscopy (PFM) measurements were conducted on a NX10 Atomic Force Microscope (Park System), equipped with a silicon tip ElectriMulti 75 (Budgetsensors). The topographical images were obtained in noncontact mode and the piezoresponse signal was obtained by applying a DC voltage ranging from −10 to 10 V in the off-field mode. Hysteresis loops were captured in the off-field mode, and a total of at least 6 cycles were used to obtain the results.

### 2.5 Electrochemical Characterization

For cyclic voltammetry assays, 5 mg mL^–1^ dispersion of BTO and BTO@PEDOT NPs were placed on top of a C11L screen-printed electrode (Metrohm DropSens), which was submerged in a deaerated 0.05% Tween 20 (Sigma-Aldrich) solution prepared in phosphate buffered saline (PBS, Sigma-Aldrich). The setup was combined with a Bipotentiostat μStat 300 (Metrohm DropSens). The assay was performed with a potential range of −0.8 to 1 V and a sensitivity of 1 × 10^–4^ A V^–1^, with scan rates of 0.01; 0.02; 0.04; 0.08; 0.1; 0.2 and 0.3 V s^–1^.

The electrical conductivity of the BTO@PEDOT NPs was measured by the four-contact probe method for at least three independent samples (*n* = 3). Initially, a suspension of BTO@PEDOT NPs was deposited on glass substrates by drop casting. Then, four gold stripes were thermally evaporated (Edwards Coating System E 306A, Irvine, CA, USA) on top of each sample. The next step involved estimating the sheet resistance (ΔV/I) of each sample by applying different electrical currents across the outer contacts using a Keithley 2400 source measure unit (Cleveland, OH, USA) and measuring the voltage drop between the inner contacts using an Agilent 34401A multimeter (Agilent Technologies, Santa Clara, CA, USA). A Veeco Dektak 8 Profilometer (Bruker, Billerica, Mass, USA) was used to measure the thickness of the samples.

### 2.6 Cell Culture

MDA-MB-231 and MCF-7 cells were cultured in RPMI Medium 1640 supplemented with 10% FBS, 1% Glutamine and 1% Penicillin/Streptomycin. HMF cells were cultured in DMEM F-12, supplemented with 10% FBS and 1% PenStrep. All cell lines were cultured at 37 °C in a humidified atmosphere with 5% CO_2_. Cell media was changed twice/thrice weekly. Cells were passaged when reaching 80% confluency.

### 2.7 Evaluation of BTO@PEDOT NPs Cytotoxicity

The evaluation of the BTO@PEDOT NPs cytotoxicity was performed using a water-soluble tetrazolium salt (WST-8) assay. The three different cell lines (MCF-7, MDA-MB-231 and HMF) were incubated at a concentration of 10^4^ cells/well in 96-well plates 24 h prior the experiment. After this, 200 and 500 nm BTO@PEDOT NPs at concentrations of 10, 25, 50, 100, 150, and 200 μg mL^–1^ were added to the culture media and incubated for further 24 h. Subsequently, media was replaced with 10% WST-8 in complete media and incubated for an hour before reading the absorbance at 450 nm in a microplate reader (Infinite 200 PRO, Tecan). Cells cultured in complete media were used as negative controls and assumed as 100% viability.

### 2.8 Determination of the Parameters of US Stimulation

HMF were cultured at a concentration of 5 × 10^5^ cells/well in a 6-well plate 24 h prior the stimulation. Different regimes were evaluated at intensities of 0.1 W cm^–2^, 0.2 W cm^–2^, 0.3 W cm^–2^, 0.4 W cm^–2^ and 0.5 W cm^–2^ for a duration of 2 min 30 s and 5 min at a fixed frequency of 1 MHz using a Sonidel SP100 Sonoporator (Sonidel, Ireland). Each condition was evaluated in triplicate (*N* = 3).

### 2.9 Live/Dead Assays

Live/dead assays were performed to assess cell viability in the presence and absence of US stimulation and BTO@PEDOT NPs. For this, after stimulation, and/or incubation with the BTO@PEDOT NPs, cells were carefully washed with PBS and incubated for 30 min in 1 μM acetoxymethyl (AM) calcein solution in PBS to stain viable cells (green) and dead cells (red) were stained with 5 μM ethidium homodimer-I in PBS. Fluorescence images were obtained with a Leica DMI3000B fluorescence microscope (Leica Microsystems). Two representative images were obtained for each condition in triplicate (*N* = 3, *n* = 2). Those were used to quantify live and dead cells using ImageJ software (ImageJ 1.51f, National Institutes of Health, Bethesda, MD, USA).

### 2.10 Reactive Oxygen Species Cytotoxicity Assay

A reactive oxygen species (ROS) detection kit based on fluorescence detection was used to identify the intracellular ROS levels in the presence/absence of US stimulation (0.4 W cm^–2^ for 2 min 30 s) and BTO@PEDOT NPs at a concentration of 200 μg mL^–1^. Cells were cultured at a concentration of 5 × 10^5^ cells/well in a 6-well plate 24 h prior the stimulation. Cells cultured in media in the absence of stimulation were used as negative controls whereas the positive control consisted of cells incubated with *tert*-butyl hydroperoxide (TBHP). The assay was performed according to the instructions of the manufacturer. The US stimulation was performed in the dark and the pictures were taken in a fluorescence microscope. Image analysis was conducted using ImageJ software, which quantified fluorescence intensity. This analysis was systematically performed at four distinct sites within each image to ensure representative sampling and accuracy in the assessment of fluorescence levels.

### 2.11 Caspase-3 Assay

After the adhesion of 1 × 10^6^ cells in 6-well plates for 24 h, MDA-MB-231 and HMF cells were cultured with 200 μg mL^–1^ BTO NPs and BTO@PEDOT NPs for 24 h, subjected to the US stimulation (0.4 W cm^–2^ for 2 min 30 s) and collected in tubes by centrifugation (1250 rpm, 5 min). 50 μL of lysis buffer was added to the cell pellet and the mixture was incubated in ice for 10 min. The tubes were again centrifuged at 10000 rpm for 1 min and each supernatant was transferred to a 96-well plate. Blanks (lysis buffer only) and controls (only cells in duplicated) were used (*N* = 3, *n* = 2). To each well, 50 μL of 2× Reaction Buffer and 0.5 μL of DTT was added, as well as 5 μL of DEVD-pNA. The microplate was incubated for 90 min at 37 °C and the output was measured in a microplate reader (Infinite 200 PRO, Tecan) at 400 nm. Caspase-3 assay relies on spectrophotometric detection of the chromophore p-nitroaniline (p-NA) after its cleavage. By comparing the absorbance of p-NA between an apoptotic sample and an untreated control, the fold increase in caspase-3 activity can be determined.

### 2.12 Membrane Potential Assay

(2–5) × 10^5^ MCF-7, MDA-MB-231 and HMF cells were seeded in 6-well plates for 24 h. Then, cells were treated with 200 μg mL^–1^ of BTO@PEDOT NPs. After further 24 h, the cells were submitted to the US treatment (0.4 W cm^–2^). To evaluate the membrane potential progression, 5 different US treatment durations were tested: no US, 30 s, 1 min 30 s, 2 min, and 2 min 30 s. After the treatment, each well was loaded with DiBAC_4_(3) in a final concentration of 500 nM. The plates were incubated protected from light with aluminum foil for 2 h and then analyzed with fluorescence microscopy, followed by their quantification using ImageJ software (*N* = 4).

### 2.13 Intracellular Calcium Quantification Assay

5 × 10^5^ MDA-MB-231 and MCF-7 cells were seeded in 6-well plates for 24 h. Then, cells were treated with 200 μg mL^–1^ of BTO@PEDOT NPs. After further 24 h, the cells were submitted to the US treatment (0.4 W cm^–2^). To evaluate the intracellular calcium progression during the treatment, 6 different US treatment durations were tested: no US, 30 s, 1 min, 1 min 30 s, 2 and 2 min 30 s. Fluo-4 AM loading solution consisted of 3 μM fluo-4 AM (reconstituted in DMSO) and 0.1% Pluronic F-127 in calcium-free PBS. To reconstitute fluo-4 AM, 44 μL of DMSO were added to one vial of fluo-4 AM (50 μg) and vortexed thoroughly. 9 μL of Pluronic F-127 were added to the reconstituted fluo-4 AM and vortexed thoroughly. Finally, 50 μL of the 860 μM fluo-4 AM/Pluronic F 127 solution were added to 14.3 mL of calcium-free PBS. Cells were washed with 1 mL of PBS, followed by the loading of 1 mL of the previous loading solution. The cells were then incubated in the dark at room temperature for 60 min and washed with 1 mL of PBS. Before the stimulation, 2 mL of PBS were added to each well, to apply the US. The cells were then analyzed with fluorescence microscopy and the fluorescence was quantified using ImageJ software (*N* = 4).

### 2.14 Cell Cycle Assay

(2–5) × 10^5^ MCF-7, MDA-MB-231 and HMF cells were seeded in 6-well plates for 24 h. 200 μg mL^–1^ BTO@PEDOT NPs were added for further 24 h. Afterward, US stimulation was performed (0.4 W cm^–2^ for 2 min 30 s). As control, cells were incubated in culture medium with 10% FBS without NPs. Cells were harvested and centrifuged at 400G for 5 min. The 1× Cell Cycle Assay Buffer and the Staining Solution were prepared according to the manufacturer’s instructions. The supernatant was removed, and the cells were washed in 2 mL ice cold 1× Cell Cycle Assay Buffer. Finally, cells were again centrifuged at 400 G for 5 min and the supernatant was removed. For the nucleic acid labeling, cells were first fixed by adding 2 mL ice cold 70% ethanol to the cell pellet and left on ice for 30 min. The cells were centrifuged at 400 G for 5 min and the supernatant was carefully removed. Cells were washed in 2 mL of 1× Cell Cycle Assay Buffer and centrifuged at 400 G for 5 min and the supernatant was carefully removed. Cells were resuspended completely with 500 μL of Staining Solution and protected from light exposure. Finally, they were incubated at room temperature for 30 min. The samples were analyzed in a FACSCalibur flow cytometer (BD Biosciences, USA). During flow cytometry data analysis, the main cell population was selected in the FSC vs SSC plot. Within the main cell population, cell debris and cell aggregates were excluded by gating on single cells in a FL2-A vs FL2-W plot. Data was analyzed using FlowJo v10 software (FlowJo LLC, USA).

### 2.15 qRT-PCR

Total RNA was extracted using the RNeasy Mini Kit (QIAGEN) following the manufacturer’s instructions. Briefly, after the treatment with BTO@PEDOT NPs and US stimulation, MDA-MB-231, MCF-7 and HMF cells were mixed with RLT lysis buffer and vortexed. Afterward, total RNA was isolated according to the manufacturer’s protocol. Total RNA was quantified using a Nanodrop (NanoVue Plus, GE Healthcare). cDNA was synthesized from the purified RNA using a High-Capacity cDNA Reverse Transcription kit. Reaction mixtures (20 μL) were incubated in a thermal cycler (96-well *T*-100 Thermal Cycler, Biorad) for 5 min at 25 °C, 20 min at 46 °C and 1 min at 95 °C and then were maintained at 4 °C. The Quantitative Reverse Transcription-Polymerase Chain Reaction (qRT-PCR) was performed using NZYSpeedy qPCR Green Master Mix (2×), ROX plus (NZYTech) and StepOnePlus real-time PCR system (Applied Biosystems). Target genes included CACNA1H and CACNA1C. Primer sequences used in the qRT-PCR analysis are presented in Table S2. All reactions were carried out at 95 °C for 10 min, followed by 40 cycles of 95 °C for 15 s and 60 °C for 1 min. All samples were analyzed in triplicates (*N* = 3, *n* = 3). Target gene expression was primarily normalized to the housekeeping gene glyceraldehyde 3-phosphate dehydrogenase (GAPDH) and then determined as a fold change relative to the baseline expression of the target gene measured in cells cultured in media in the absence of stimulation (control conditions).

### 2.16 Statistical Analysis

Data were expressed as mean ± standard deviation (SD). Statistical significance was performed by one-way or two-way ANOVA using GraphPad Prism v10.1.1. When *p* < 0.05 data were considered statistically significant (*p < 0.05, **p < 0.01, ***p < 0.001 and ****p < 0.0001)

## 3 Results and Discussion

### 3.1 Characterization of BTO@PEDOT NPs

**Figure 1:**
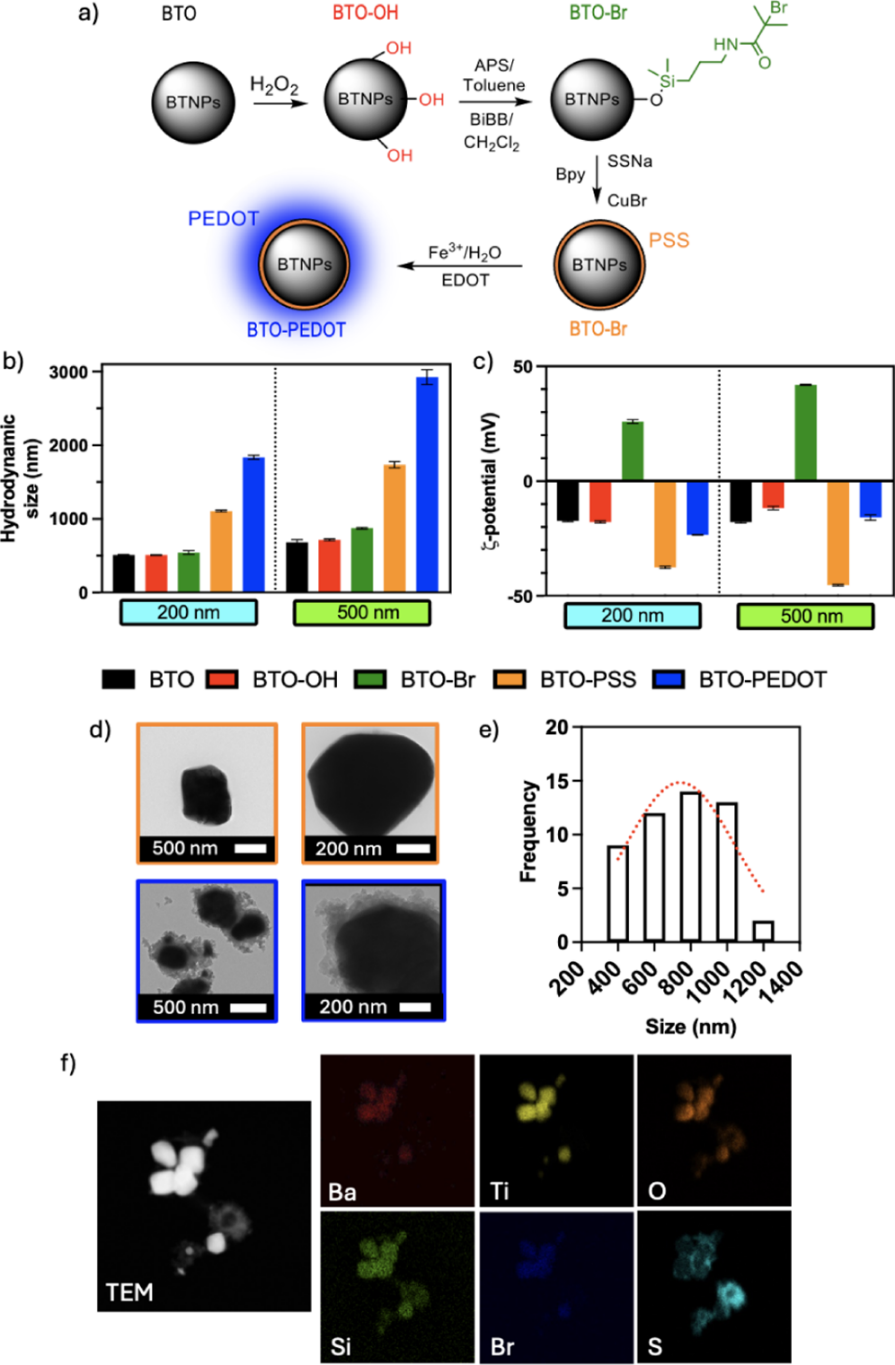
Synthesis and characterization of BTO@PEDOT NPs. a) Schematic representation of the synthesis of the nanobioelectronic systems proposed in this work to obtain the BTO@PEDOT NPs.^30^ b) Hydrodynamic size and c) ζ-potential of the different intermediates of the synthesis of BTO@PEDOT NPs obtained by Dynamic-light scattering for the 200 and 500 nm BTO (*n* = 3). d) TEM images of the 500 nm BTO-PSS (orange) and BTO@PEDOT NPs (blue). e) Size distribution (*n* = 50) and f) elemental mapping of the 500 nm BTO@PEDOT NPs.

The synthesis of BTO@PEDOT NPs consisted of a four-step protocol^30^ (Figure 1a): (i) hydroxylation, (ii) silanization, (iii) surface initiated atomic transfer radical polymerization (SI-ATRP) and (iv) oxidative chemical polymerization to produce BTO@PEDOT NPs. Two modifications were introduced from the original synthesis: 1) we explored two different BTO sizes (200 and 500 nm), in contrast to the previous work^30^ where only 200 nm BTO were used; and 2) different PSS:BTO ratios (1.5:1, 2.5:1 and 3.5:1) were assessed in the ATRP step. We hypothesized that higher BTO sizes could potentially amplify better the electrical output provided by the US as shown in other works^43^ as this effect has not been demonstrated in vitro. It is also important to note that the BTO@PEDOT NPs formulation was optimized iteratively, based on the outcomes of the cellular studies described in the following section. In subsequent experiments, the BTO@PEDOT NPs used correspond to the 500 nm BTO and 1.5:1 PSS:BTO ratio unless otherwise stated.

The success of the BTO@PEDOT NPs synthesis was confirmed through several techniques, namely Fourier Transform infrared analysis (FTIR), Dynamic Light Scattering (DLS), Transmission Electron Microscopy (TEM) and Energy dispersive X-ray (EDX). DLS measurements were performed for the optimized PSS:BTO ratio on the 200 and 500 nm BTO. DLS showed that the BTO@PEDOT NP size increased while the zeta potential changed dynamically following each synthesis step, indicating that surface modifications were taking place. The initial hydrodynamic size corresponded to 478.7 and 682.2 nm, respectively, increasing after each chemical modification and reaching 1836.6 and 2925 nm (Figure 1b). Values of zeta potential (ζ) also changed with the functional group additions and polymer layer formations on the BTO from −17.7 mV and −11.7 mV in BTO–OH NPs, to 25.9 mV and 41.9 mV in BTO-Br NPs, to values of −37.5 mV and −45.3 mV after PSS modification, and finally −23.4 mV and −15.8 mV in the BTO@PEDOT NPs (Figure 1c).

FTIR analysis of naked BTO revealed peaks at 1455 cm^–1^, 1634 and 3412 cm^–1^ corresponding to the stretching vibration of −CO_3_^2–^, associated with barium carbonate, precursor in BTO synthesis, and stretching in Ti–O (normal and bending mode), Ti–OH and Ba–OH bonds in BTO, as reported in previous works^44−46^ (Figure S1). A peak associated with the hydroxylation step (BTO–OH) was also observed at 1638 cm^–1^, corresponding to the bending mode of H–O–H.^46^ Regarding the BTO-Br, absorption peaks at 1133 cm^–1^ (Si–O-particle) and 1034 cm^–1^ (Si–O–Si) were observed, suggesting that silane was successfully attached to the surface of BTO. A broad peak between 2851 and 2090 cm^–1^ that might be attributed to the N–H stretching, present between the Si and the α-bromoisobutyryl bromide group, could also be observed.^30^ Peaks at 1298 and 1194 cm^–1^ corresponding to the S=O stretching vibrations from sulfonic acid groups, indicate the presence of PSS. The two peaks between 1000 and 1100 cm^–1^ can be attributed to aromatic C–H in-plane bending and C–S stretching vibrations, which are also characteristic of PSS.^47,48^ The peak at 1526 cm^–1^ is usually associated with C=C stretching vibrations from the thiophene ring in PEDOT and the peaks at 834 cm^–1^, 920 and 978 cm^–1^ are characteristic of the C–S stretching vibrations in PEDOT, which confirms the successful grafting of the PEDOT shell.

Further characterization of the BTO-PSS and BTO@PEDOT NPs was performed by TEM and EDX. Morphological differences after PSS grafting and PEDOT polymerization are presented in Figure 1d. On the top images, a light outer layer grafted to the core can be observed and attributed to the grafted PSS shell layer. A higher magnification image of this layer is presented in Figure S2, with an approximate thickness of 50 nm. After the polymerization of the PEDOT, a cloud-like structure is observed on the bottom images around the core. The size distribution of the particles was calculated from the TEM images, with diameters in the range 400–1200 nm (Figures 1e and S3) which might be related to the polydispersity of the initial BTO and uneven polymer growth. EDX analysis of BTO@PEDOT NPs revealed a high content of Ba, Ti and O at the core, as expected, and a sulfur corona around it that could be attributed to the PEDOT (Figure 1f). Additionally, the elemental mapping revealed a heavy presence of Si at the core, stemming from the silanization step, and Br from the ATRP initiation step. Traces of Cu, catalyzer of the PSS grafting, can also be observed at the core, and Fe is also present at the PEDOT shell as it is used as catalyzer and doping agent (Figure S4).

**Figure 2:**
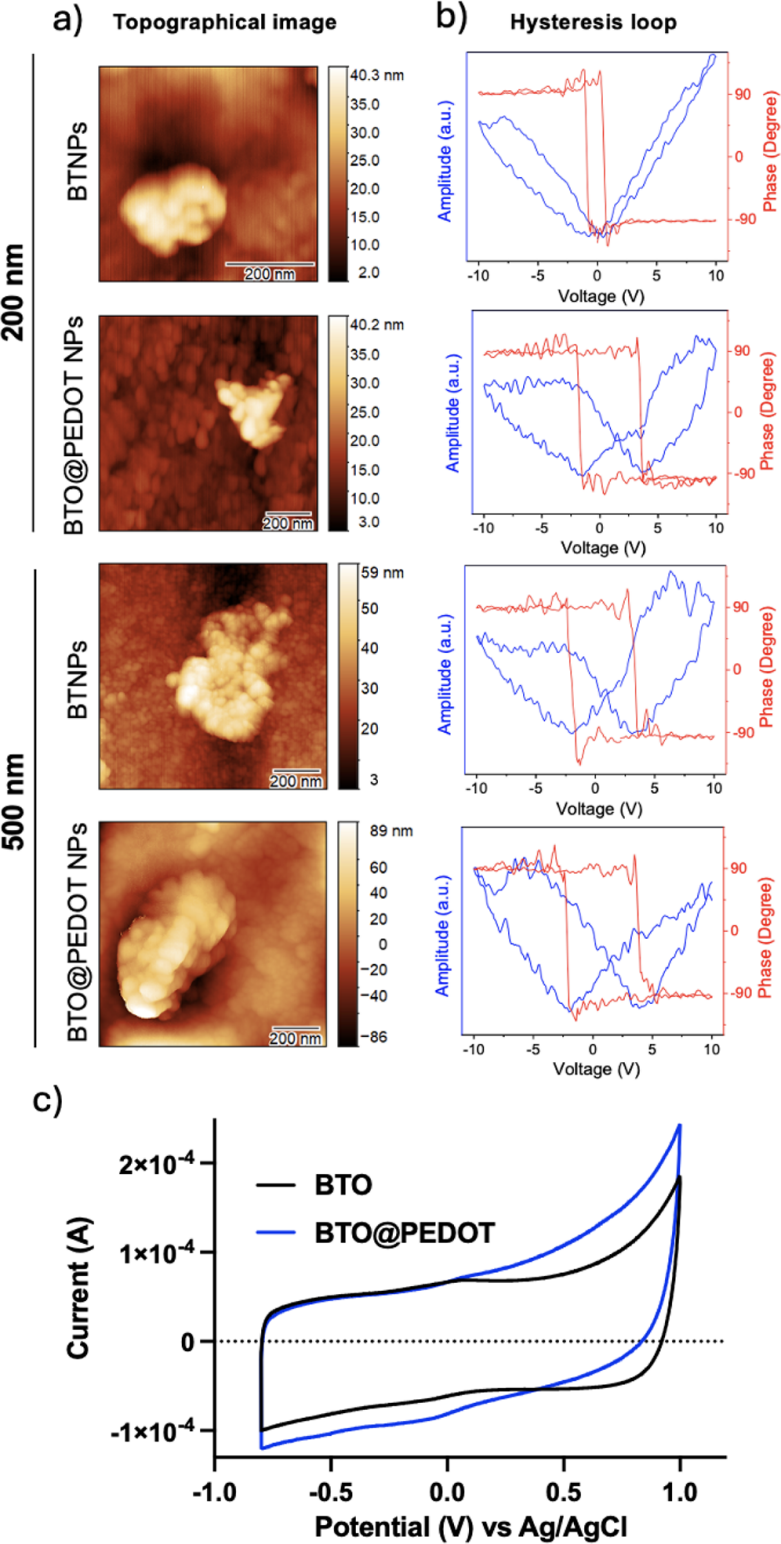
Piezoelectric and electrochemical analysis of BTO@PEDOT NPs. a) Topographical image of an individual nanoparticle and b) its representative hysteresis loop illustrating the piezoresponse behavior of the material. The amplitude and phase of the piezoresponse are plotted against the DC applied voltage. c) Cyclic voltammetry curves for BTO, and BTO@PEDOT NPs at 300 mV/s scan rate.

The evaluation of the potential mechanoelectrical transduction of the BTO@PEDOT NPs was performed indirectly by piezoelectric Atomic Force Microscopy (piezoAFM), as direct measurement under US is challenging due to the lack of specialized instrumentation and the nanoscale size of the particles. The piezoelectric nature of the NPs was confirmed due to the 180° phase shift and the amplitude butterfly shape in the hysteresis loops (Figures 2a,b). Furthermore, a higher BTO size and the presence of PEDOT are associated with increased coercivity (Vc) requiring a larger applied voltage for their poling (Vc_BTO 200 nm_ = 0.59 ± 0.12; Vc_BTO@PEDOT 200 nm_ = 1.84 ± 0.38; Vc_BTO 500 nm_ = 2.05 ± 0.46; Vc_BTO@PEDOT 500 nm_ = 2.54 ± 0.44).

To further validate the enhancement of electrical properties caused by the PEDOT layer, we performed a four-contact probe assay on drop-casted 500 nm BTO@PEDOT NPs, which revealed a conductivity of 3.08 × 10^–5^ S cm^–1^. Other works on plain PEDOT NPs have reported similar conductivity values, namely 2.1 × 10^–6^ S cm^–1^.^46,49^ In contrast, BTO displayed insulating behavior, with no measurable conductivity, underscoring a significant improvement conferred by the PEDOT coating.

Cyclic voltammetry (CV) was also performed to understand the electrochemical behavior and stability of BTO and BTO@PEDOT NPs. The CV curves exhibit quasi-rectangular shapes suggesting an electrical double layer capacitive behavior and charge storage capability of the materials, which increases with the addition of the PEDOT layer (Figure 2c). This shape is also observed at different scan rates (Figure S5). A very small peak can also be observed in the BTO at 0.067 V and −0.050 V, becoming less evident in the BTO@PEDOT NPs (0.084 V and −0.080 V). Nevertheless, these results demonstrate that the particles are electrochemically stable.

### 3.2 US Optimization and Preliminary Assessment of Cell Viability

The biocompatibility of the 200 and 500 nm BTO@PEDOT NPs was evaluated using a WST-8 metabolic activity assay as shown in Figure S6 on the two breast cancer cell lines MCF-7 and MDA-MB-231 and the healthy model consisting of primary human mammary fibroblasts (HMF). From those results it was possible to observe that concentrations up to 200 μg mL^–1^ did not impact cell viability in any of the cell lines.

Parameters of US stimulation were then optimized on HMF cells to avoid a detrimental effect on cell viability in the absence of the BTO@PEDOT NPs. No morphological differences were observed after stimulation (Figure S7), however, longer exposure to the US, particularly at higher intensities, seemed to negatively impact HMF viability (Figure S8). Nevertheless, viability values were above 91.07% up to an intensity of 0.4 W cm^–2^, and for subsequent experiments the US stimulation parameters consisted of 0.4 W cm^–2^ for 2 min 30 s.

The formulation of the particles was optimized according to the effect they displayed in the viability of MCF-7 cells prior and after US stimulation. For 200 nm BTO, the PSS:BTO ratio of 1.5:1 showed a reduction in cell viability with US stimulation but was considered insufficient for this application (Figure S9). The 2.5:1 ratio was more effective but also exhibited cytotoxicity in the absence of US, leading to its rejection (Figure S9). The 3.5:1 ratio did not significantly affect viability upon stimulation; thus, it was also discarded. In contrast, for 500 nm BTO, the PSS:BTO ratio of 1.5:1 effectively reduced MCF-7 viability in a dose-dependent manner without cytotoxic effects, making it the preferred formulation in subsequent studies (Figure S10). The 2.5:1 ratio of 500 nm BTO was discarded due to similar cytotoxic concerns, and the 3.5:1 ratio caused particle aggregation, rendering it unsuitable.

### 3.3 Antitumor Effects of US-Stimulated BTO@PEDOT NPs

Once the US stimulation regime and the BTO@PEDOT formulation was selected, the antitumor effects of the BTO@PEDOT NPs under external US stimulation were evaluated on the MCF-7, MDA-MB-231 and HMF cell lines.

**Figure 3:**
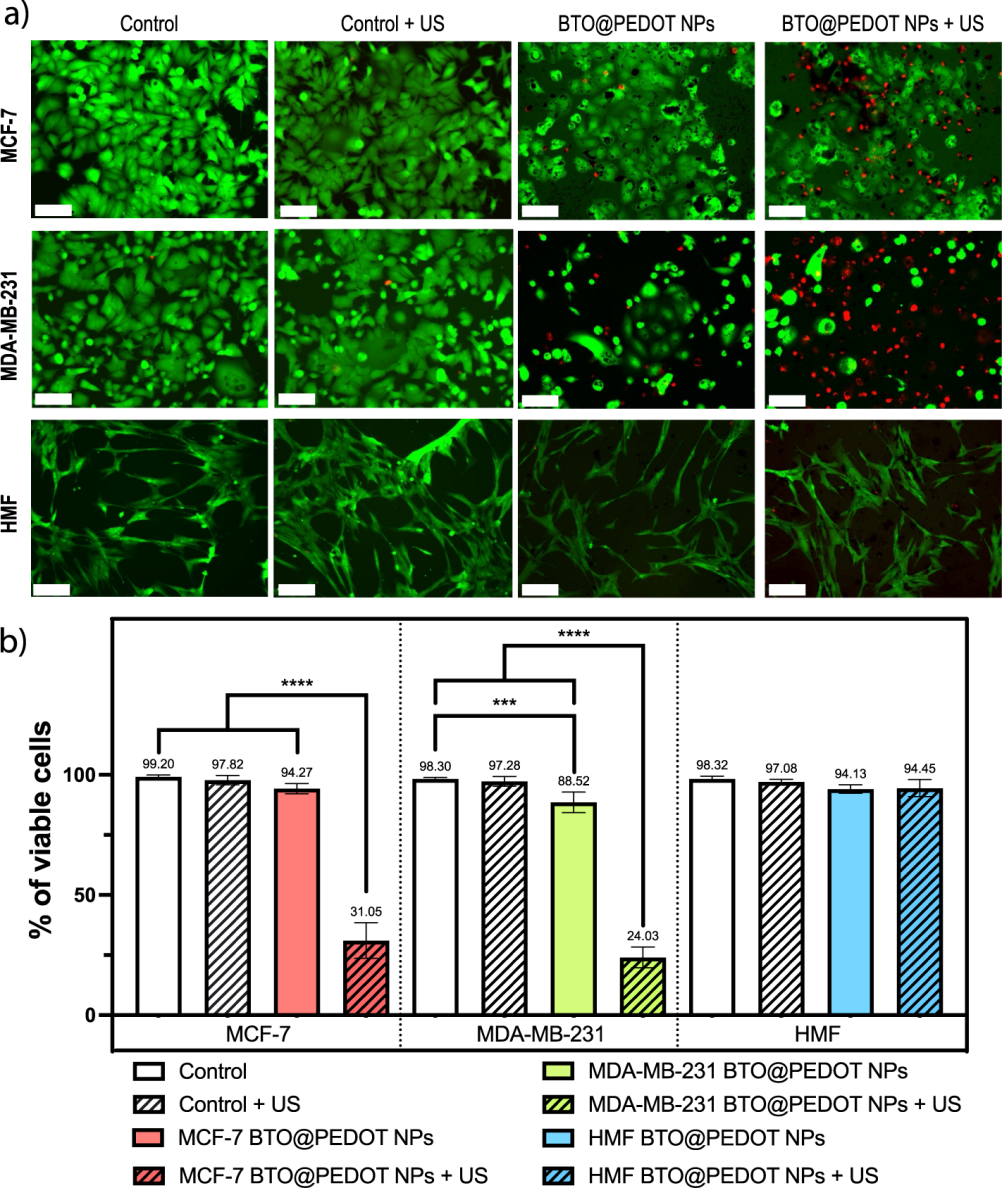
Treatment consisting of BTO@PEDOT NPs and US stimulation decreases the viability of human breast cancer cell lines. a) Representative fluorescence microscopy images of the MCF-7, MDA-MB-231 and HMF cells in the absence (controls) and presence of BTO@PEDOT NPs at a concentration of 200 μg mL-1 and ultrasound (US) stimulation (I = 0.4 W cm^–2^, *f* = 1 MHz, DC = 50%) after live/dead staining. Scale bar 100 μm. Green fluorescence was acquired through cell-permeant dye calcein AM and red fluorescence was acquired through the high-affinity nucleic acid stain ethidium homodimer. b) Calculation of the percentage of viable cells at the previous conditions. Statistical analysis was performed using one-way ANOVA (***p < 0.001 and ****p < 0.0001). Data are shown as mean ± SD (*N* = 3, *n* = 2).

As shown in Figure 3a,b, US on its own does not lead to any significant cytotoxic effects in any of the cell lines. However, when the BTO@PEDOT NPs are incubated with the cells in the absence of US, there is a slight decrease in viability in the MDA-MB-231 cells, reaching 88.52%. This was not observed in the MCF-7 or the HMF cells, where they maintained values of 94.27% and 94.13%, respectively. Interestingly, when US stimulation is applied in the presence of the BTO@PEDOT NPs, there is a profound and significant decrease in the viability of both cancer cell lines, reaching 31.05% in MCF-7 and 24.03% in MDA-MB-231. In the case of the HMF, cell viability is maintained at 94.45% (Figure 3b).

### 3.4 Evaluation of the Cancer Bioelectrical Dysregulation

**Figure 4:**
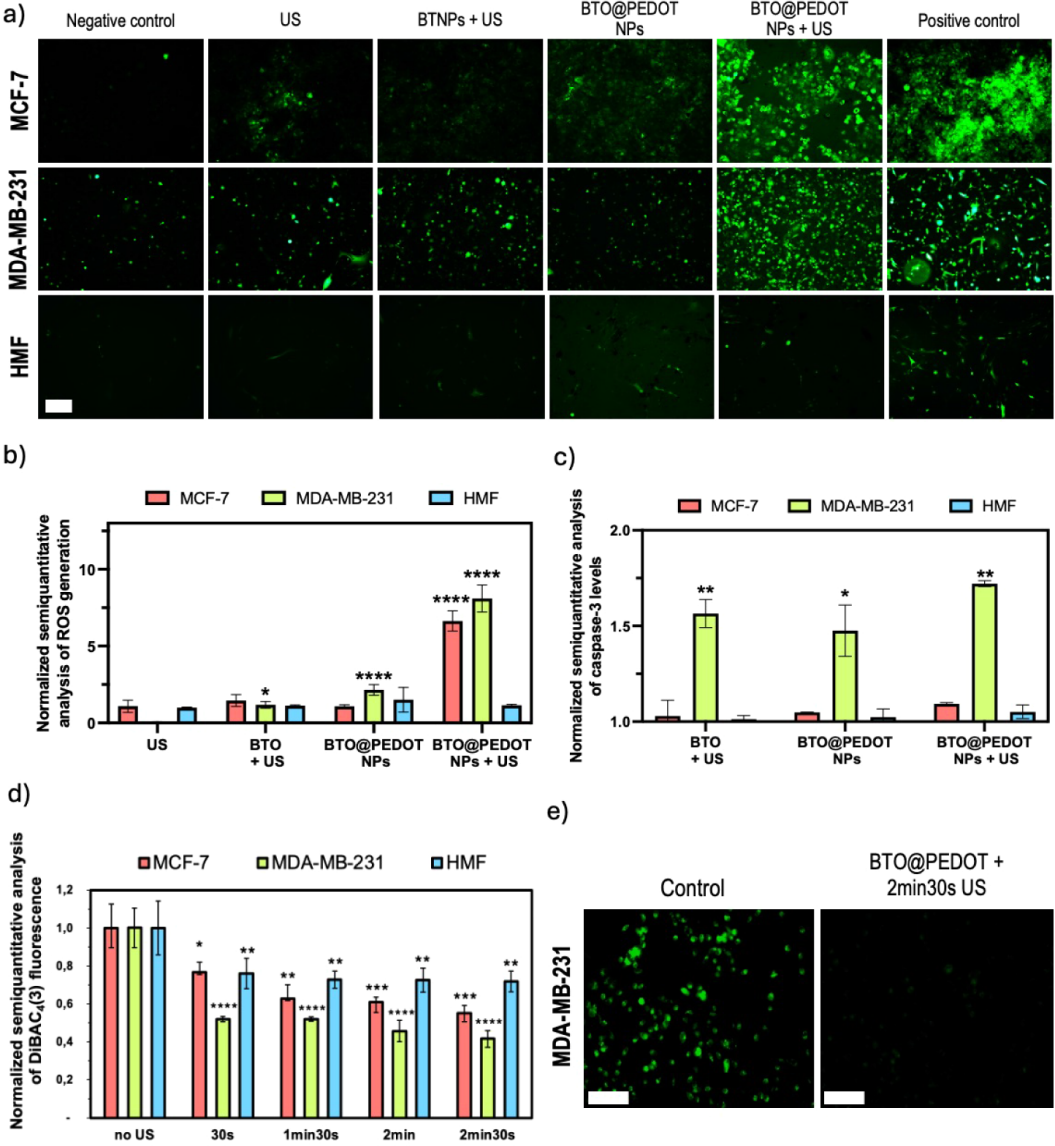
US stimulation in the presence of BTO@PEDOT NPs increases ROS production in MCF-7 and MDA-MB-231, activates caspase-3 in MDA-MB-231 cells and polarizes cell membranes. a) Fluorescence microscopy images of ROS generation and normalized semiquantitative analysis of b) ROS generation (*N* = 4) and c) caspase-3 activity (*N* = 3, *n* = 2) in MCF-7, MDA-MB-231 and HMF cells under different conditions. d) Normalized semiquantitative analysis of DiBAC_4_(3) levels in MCF-7, MDA-MB-231 and HMF cells under different US application times and with BTO@PEDOT NPs (*N* = 4). e) Fluorescence microscopy images of control and US-stimulated MDA-MB-231 in the presence of BTO@PEDOT NPs after evaluation with DiBAC_4_(3), which correlates higher cell membrane polarization with lower fluorescence. Scale bar 200 μm. . Statistical analysis was performed using two-way ANOVA (*p < 0.05, **p < 0.01, ***p < 0.001 and ****p < 0.0001). Data are shown as mean ± SD.

MDA-MB-231 cells inherently exhibited higher baseline ROS levels than the MCF-7, as described in other studies^50,51^ (Figure 4a). For both cancer cell lines, baseline levels of ROS are observed to be higher than those in HMF cells. When US stimulation was applied, a slight increase in ROS production was observed in all conditions. The application of US in the presence of naked BTO was also evaluated, resulting in a significant increase only in MDA-MB-231 cells (Figure 4b). This further confirms the critical role of the PEDOT layer in enhancing the cytotoxic effects of BTO@PEDOT NPs, as naked BTO are not as effective (Figure S11). When cells were stimulated with US in the presence of the BTO@PEDOT NPs, a marked 6-fold and 8-fold increase in ROS production, equivalent to 371 μM and 281 μM of TBHP-induced toxicity based on a fluorescence-TBHP calibration curve (Figure S12), was observed in MCF-7 and MDA-MB-231, respectively, while no significant increase was observed in the HMF (Figure 4b). TBHP is a lipid hydroperoxide analogue commonly used as a pro-oxidant in vitro. It mimics the toxic effects of peroxidized fatty acids, leading to oxidative stress by damaging DNA, lipids, and other macromolecules.^52^ Previous studies have shown that concentrations starting at 100 μM of TBHP may be toxic to breast cancer cells.^53^ In cancer cells, higher ROS levels are linked to increased metabolic activity, oncogene expression, and mitochondrial dysfunction, promoting cell proliferation, survival, and metastasis.^54^ Yet, excessive ROS levels can also be detrimental to cancer cells, leading to oxidative stress, cell damage, and apoptosis.^55^ Although ROS is commonly used as a toxic agent to target cancer cells in many treatments, we suspected that other mechanisms related to bioelectric disruption might also drive toxicity in our system. To explore this possibility, additional assays were performed to understand the roles of membrane depolarization, intracellular calcium changes, and cell cycle arrest in mediating the observed effects.

To achieve this, we quantified caspase-3 levels in all cell lines with and without US stimulation, as caspase-3 plays a pivotal role in orchestrating apoptosis.^56,57^ We observed a 1.7-fold increase in caspase-3 levels of US stimulated MDA-MB-231 cells in the presence of the BTO@PEDOT NPs (Figure 4c). However, significantly increased levels of caspase-3 were also observed in unstimulated conditions in the presence of BTO@PEDOT NPs and when those cells were stimulated in the presence of BTO. In the case of MCF-7 and HMF cells, caspase levels did not significantly increase. Diminished caspase activity typifies most cancer cells, as they lack the standard mechanisms for programmed cell death, contributing to their uncontrolled proliferation.^58^ Our results showed that MDA-MB-231 cells exhibited significantly higher caspase-3 activity when compared with MCF-7 cells upon treatment, which are inherently caspase-3 deficient, and could be linked to their higher susceptibility to the treatment.^59^

A membrane potential assay was performed to evaluate the capacity of the treatment to polarize cancer cells. Our results revealed increased polarization with increasing times of US application on MCF-7, MDA-MB-231 and HMF cells with BTO@PEDOT NPs, evidenced by a significant decrease in fluorescence intensity (Figure 4d,e). This polarization can disrupt cancer cells’ bioelectric mechanisms, leading to their death and cell cycle arrest, since quiescent states are associated with membrane hyperpolarization,^60^ underscoring the role of bioelectricity as a fundamental determinant in the pathophysiology of cancer progression. US application on its own did not polarize cell membranes; in fact, in MDA-MB-231 cells, it caused a slight depolarization (Figure S13). Mechanical stimuli can activate Piezo channels, which are overexpressed in more aggressive cancers, increasing intracellular calcium influx and potentially enhancing tumorigenic properties, correlated with higher depolarization.^61,62^

**Figure 5:**
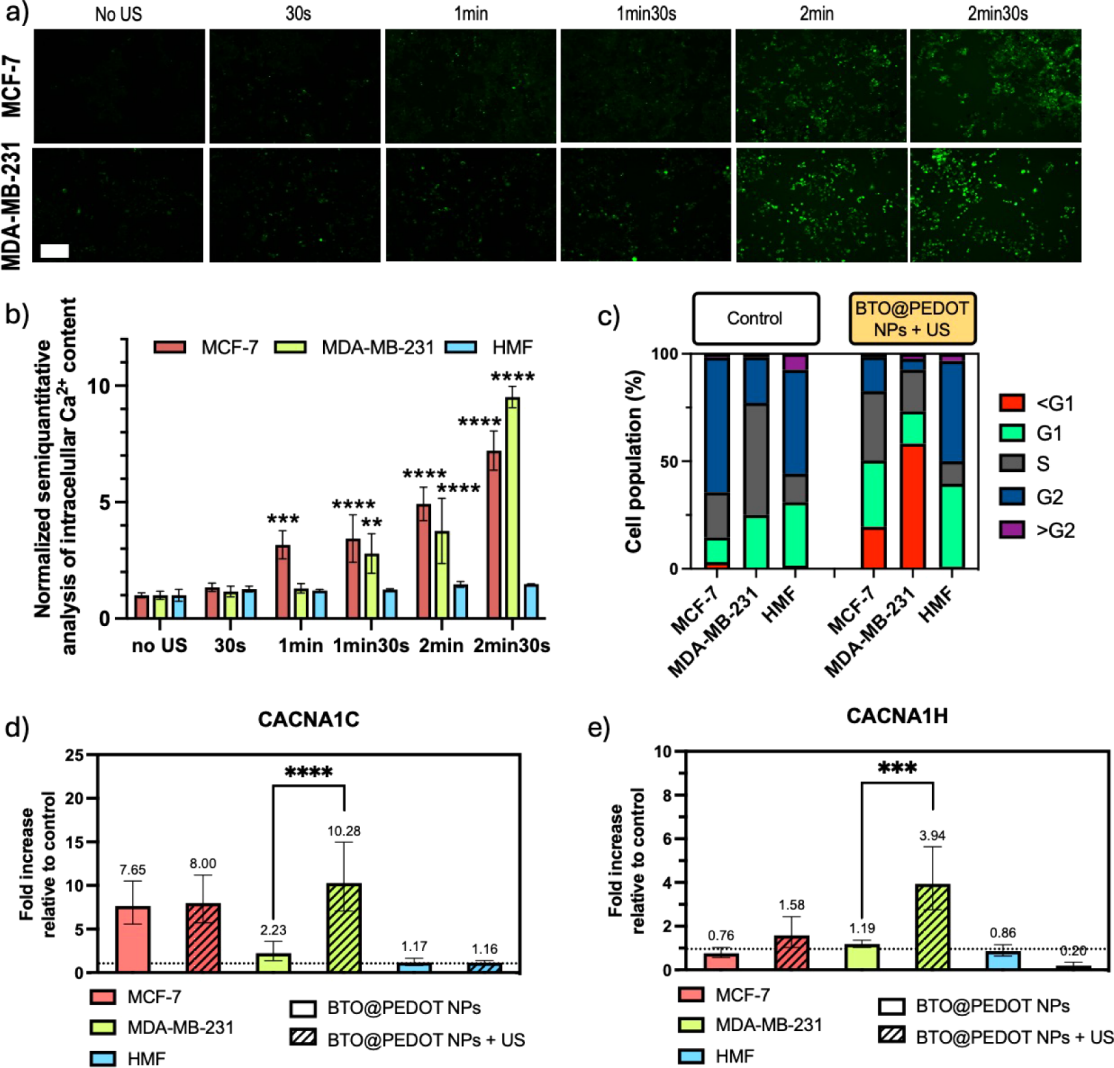
US stimulation in the presence of BTO@PEDOT NPs increases intracellular Ca^2+^in MCF-7 and MDA-MB-231 cells, alters their cell cycle and upregulates CACNA1C and CACNA1H genes in MDA-MB-231 cells. a) Fluorescence microscopy images and b) normalized semiquantitative analysis of the intracellular Ca^2+^ content of MCF-7, MDA-MB-231 and HMF cells at increasing times of US stimulation in the presence of BTO@PEDOT NPs. Scale bar 200 μm. Statistical analysis was performed using two-way ANOVA (*p < 0.05, **p < 0.01 and ****p < 0.0001). Data are shown as mean ± SD (*N* = 4). c) Cell cycle analysis of MCF-7, MDA-MB-231 and HMF cells. Percentage of cells in each phase of the cell cycle as determined by fluorescence-activated cell sorting (FACS) analysis in control conditions and after US stimulation in the presence of BTO@PEDOT NPs. d) CACNA1C and e) CACNA1H gene expression of obtained by RT-qPCR analysis of MCF-7, MDA-MB-231 and HMF cells incubated with BTO@PEDOT NPs with and without US stimulation. Gene expressions are normalized against the housekeeping gene GAPDH and presented as fold-change levels relative to controls consisting on cells cultured on media (represented by dotted line) (*N* = 3, *n* = 3). Statistical analysis was performed using one-way ANOVA (***p < 0.001 and ****p < 0.0001). Data are shown as mean ± SD.

Following this, the quantification of intracellular Ca^2+^ was performed due to its involvement in various bioelectrical processes^63^ as the modulation of Ca^2+^ influx is involved in cellular signaling pathways. For instance, voltage-gated Ca^2+^ channel antagonists, such Mibefradil,^64,65^ inhibits breast cancer progression and invasion.^66^ When MCF-7 and MDA-MB-231 cells were stimulated with US in the presence of the BTO@PEDOT NPs, a time-dependent increase in intracellular Ca^2+^ was observed (Figure 5a), reaching a 7.2 and 9.5-fold increase in MCF-7 and MDA-MB-231 cells, respectively after 2 min 30 s of stimulation (Figure 5b). US in the absence of the BTO@PEDOT NPs can slightly increase intracellular Ca^2+^ levels, a finding supported by previous literature which suggests that mechanical stimuli like US can induce transient Ca^2+^ influx through mechanosensitive pathways, including Piezo1 channels and other stretch-activated channels, contributing to various cellular responses (Figure S14).

Since cancer cells also exhibit enhanced proliferative and survival capabilities under depolarized conditions,^67^ we also evaluated the potential of the BTO@PEDOT NPs to interfere with the cell cycle. Our results show a reduction in the number of MCF-7 cells in the G2 phase from 62.6% to 15.9% after US stimulation of the BTO@PEDOT NPs, and from 21.2% to 5.12% in MDA-MB-231 cells (Figure 5c). It is well-known that the membrane potential fluctuates during the cell cycle. At the G1/S border, Vm undergoes hyperpolarization. During the G2 and M phases, Vm depolarizes, and quiescent cells in the G0 stage exhibit mitotic activity after Vm depolarization. The polarization caused by the stimulation of BTO@PEDOT NPs may hinder cells from entering the depolarized G2 phase, as shown in the assay, causing them to arrest at G1 and stop their mitotic activity. In the case of the HMF, values remain uniform consisting of 48.3% and 46.6%. This contrasts with the increase in the sub-G1 (<G1) population of MCF-7 and MDA-MB-231 cells, an indicator of apoptotic events,^68^ after US stimulation of BTO@PEDOT NPs, where values increase from 3.15% to 19.5% and from 0.32% to 58.2%, respectively (Figure 5c). The percentage of HMF cells at the sub-G1 phase does not vary significantly, consisting of 1.43% and 0.95%. The previous results were calculated from the graphs obtained by fluorescence-activated cell sorting (FACS) shown in Figures S15–S17. These bioelectric disruptions, alongside the increased intracellular calcium signaling—a key bioelectric parameter—provide consistent evidence that our nanobioelectronic system can harness cancer’s bioelectricity. Previous works also demonstrated that electrical stimulation led to an increase in intracellular calcium content,^69−71^ further corroborating our data.

We also evaluated the impact of the US stimulation of the BTO@PEDOT NPs on the expression of two voltage dependent Ca^2+^ channels associated with breast cancer progression, encoded by the genes CACNA1C and CACNA1H. In the case of MDA-MB-231, there is a 10.28-fold increase in the expression of CACNA1C after US stimulation in the presence of BTO@PEDOT NPs relative to controls (Figure 5d), significantly increasing from the 2.23-fold increase observed in unstimulated conditions, aligning with the results obtained for intracellular Ca^2+^ content. However, in MCF-7 cells, data is inconclusive, as the BTO@PEDOT NPs presence is correlated with increased CACNA1C expression, regardless of stimulation—7.65-fold higher unstimulated and 8-fold when stimulated, compared to controls. In HMF cells, CACNA1C levels remained comparable to controls. Similarly, MDA-MB-231 cells showed a marked upregulation of CACNA1H upon stimulation in the presence of BTO@PEDOT NPs, with an increase from 1.19-fold to 3.94-fold, while MCF-7 and HMF cells maintained stable levels in all conditions (Figure 5e). The CACNA1C gene encodes an L-type voltage-dependent calcium channel associated with cancer cell proliferation,^72^ cell cycle progression^73^ and invasion,^66^ while CACNA1H encodes a T-type CaV3.2 member of the alpha-1 subunit family, a protein in the voltage-dependent calcium channel complex. These results suggest that BTO@PEDOT NPs could potentially induce the upregulation of the CACNA1C and CACNA1H genes in MDA-MB-231 cells, which are essential to maintain their bioelectrical activity and possibly modulate their metastatic and aggressive behavior. The fact that the MDA-MB-231 cells have the potential to easily upregulate these channels, might stem from their metastatic and aggressive phenotype, naturally allowing the cells to produce more voltage-gated calcium channels.

## 4 Conclusions

In this work, a wireless nanobioelectronic system activated by external US was developed to effectively harness cancer’s bioelectrical properties. BTO@PEDOT NPs upon US stimulation were able to significantly reduce the viability of MCF-7 and MDA-MB-231 cells, while BTO@PEDOT NPs and US on their own did not impact cell viability. Our healthy model consisting of HMF was not affected, suggesting that cancer cells present a higher susceptibility to this treatment, representing a novel strategy toward cancer therapeutic intervention.

Other systems based on piezoelectric NPs require more intense and/or frequent stimulation regimes to achieve the same results on cancer cell viability,^69^ which could increase the risk of side effects such as heating and cavitation of the NPs. Our US stimulation parameters, on the other hand, lay within the clinical range (1.0 MHz, 0.4 W cm^–2^) as frequencies used in therapy are typically between 1.0 and 3.0 MHz and intensities often below 1 W cm^–2^. This also demonstrates the improved ability of the BTO@PEDOT NPs over other piezoelectric NPs to transduce external US stimulation.

We hypothesize that our nanobioelectronic system is capable of rewiring the bioelectric circuitry of cancer cells, targeting their inherent electrical dysregulation and that bioelectricity is a much more critical driver of cancer cell behavior than is currently understood. In this study, we observed that our treatment effectively polarized the membranes of cancer cells, which are known to have more depolarized membranes compared to healthy cells, particularly the MDA-MB-231 cell line. Previous work has established that MDA-MD-231 cells present a higher electrical activity than the nonmetastatic cell line MCF-7, which could be linked to its aggressiveness and metastatic potential.^74^ This led to a significant increase in cell death, suggesting that these cells, due to their bioelectrical abnormalities, may rely heavily on their dysregulated bioelectric states to maintain their aggressive behavior. Additionally, we demonstrated that the cell cycle was halted, with a marked decrease in cells in the G2 phase—a phase that also requires depolarization. This polarization was associated with increased ROS production, elevated intracellular calcium levels, and overexpression of voltage-gated calcium channels. In contrast, healthy HMF cells, which maintain polarized cell membranes and do not rely as heavily on bioelectricity for homeostasis, were largely unaffected by the treatment.

While this study serves as a proof of concept for the potential of BTO@PEDOT NPs in cancer therapy, future efforts will include optimizing synthesis methods to be more environmentally friendly and sustainable. By adopting greener synthesis approaches, we aim to further improve the safety profile of our nanomaterials for potential clinical applications.

These findings not only contribute to expanding our comprehension of the bioelectrical aspects of cancer but also pave the way for the development of advanced noninvasive therapeutic strategies that exploit the bioelectrical susceptibility of cancer cells.

## Supporting Information Available

Reagent quantities used in the synthesis of the different PSS:BTO ratios (Table S1); primer sequences used for qRT-PCR analysis (Table S2); FTIR characterization of the BTO, BTO–OH and BTO-Br and BTO-PSS intermediaries and BTO@PEDOT NPs (Figure S1); high-resolution TEM image of BTO-PSS particle (Figure S2); TEM image of 500 nm BTO@PEDOT NPs (Figure S3); Cu and Fe distribution on the BTO@PEDOT NPs analyzed by EDX (Figure S4); cyclic voltammogram of the BTO and BTO@PEDOT NPs at different scan rates (Figure S5); WST-8 metabolic activity assay in all cell lines incubated with increasing concentrations of 200 and 500 nm BTO@PEDOT NPs (Figure S6); fluorescence microscopy images of the HMF after application of different parameters of US (Figure S7); calculation of the HMF viability after US stimulation (Figure S8); percentage of viable MCF-7 cells at increasing concentration of 200 and 500 nm BTO@PEDOT NPs with different PSS:BTO ratios (Figures S9–S10); percentage of viable MCF-7 cells at increasing concentration of 500 nm BTNPs (Figure S11); calibration curve of ROS fluorescence measured at increasing concentrations of TBHP (Figure S12); DiBAC_4_(3) fluorescence detected after US stimulation (Figure S13); intracellular calcium concentrations obtained after US stimulation (Figure S14); evaluation of the cell cycle of MCF-7, MDA-MB-231 and HMF cells performed by flow cytometry (Figures S15–S17) (PDF)
