# Supplementary material for: Wireless Stimulation of Barium Titanate@PEDOT Nanoparticles Toward Bioelectrical Modulation in Cancer
Source: ACS Appl Mater Interfaces. 2025 Jan 29;17(6):8836–48. doi: 10.1021/acsami.4c12387 (PMC11827599; doi:10.1021/acsami.4c12387)
Supplement: Supplementary file 1 — am4c12387_si_001.pdf [file am4c12387_si_001.pdf]

Supporting information

Wireless Stimulation of Barium Titanate@PEDOT Nanoparticles towards Bioelectrical Modulation in Cancer

Catarina Franco Jones[1,2], Marta S. Carvalho[1,2], Akhil Jain[3], Paula Rodriguez-Lejarraga[4], Filipa Pires[5], Jorge Morgado[5], Senentxu Lanceros-Mendez[4,6,7], Frederico Castelo Ferreira[1,2,], Teresa Esteves[1,2,*], Paola Sanjuan-Alberte[1,2,*]*

[1] Department of Bioengineering and iBB - Institute of Bioengineering and Biosciences, Instituto Superior Técnico, Universidade de Lisboa, Av. Rovisco Pais, 1049-001 Lisbon, Portugal

[2] Associate Laboratory i4HB—Institute for Health and Bioeconomy, Instituto Superior Técnico, Universidade de Lisboa, Av. Rovisco Pais, 1049-001 Lisbon, Portugal

[3] Division of Pharmacy and Optometry, Faculty of Biology, Medicine and Health, University of Manchester, Oxford Road, Manchester, M13 9TL, UK

[4] BCMaterials, Basque Center for Materials, Applications and Nanostructures, UPV/EHU Science Park, 48940 Leioa, Spain

[5] Department of Bioengineering and Instituto de Telecomunicações (IT), Instituto Superior Técnico, Universidade de Lisboa, 1049-001 Lisboa, Portugal

[6] Centre of Physics Universities of Minho and Porto (CFUM-UP), University of Minho and Laboratory of Physics for Materials and Emergent Technologies, LapMET, Campus de Gualtar, 4710-057 Braga, Portugal

[7] Ikerbasque, Basque Foundation for Science, 48009 Bilbao, Spain

*E-mail: frederico.ferreira@tecnico.ulisboa.pt, teresa.esteves@tecnico.ulisboa.pt, paola.alberte@tecnico.ulisboa.pt

Table S1. Reagents quantities used in the synthesis of the three PSS:BTO ratios

**PSS:BTO**	**NaSS (g)**	**EDOT (mL)**	**$FeCl_3$ (g)**
1.5:1	3.375	0.225	0.3
2.5:1	5.625	0.375	0.5
3.5:1	7.875	0.525	0.7

BTO: barium titanate, EDOT: 3,4-Ethylenedioxythiophene, NaSS: Sodium styrene sulfonate, PSS: Polystyrene sulfonate

Table S2. Sequences of primers used for qRT-PCR analysis

**Gene**	**Sequence**
GADPH	Fwd: 5’-GGTCACCAGGCTTTTA-3’<br>Rev: 5’-CCTGGAAGATATGGGA-3’
CACNA_1H	Fwd: 5’-GGAACATCTCCACCAAGGCACA-3'<br>Rev: 5’-TCCATCCTTGGATGACAGCACG-3’
CACNA_1C	FWD: 5’-GCAGGAGTACAAGAACTGTGAGC-3’<br>Rev: 5’-CGAAGTAGGTGGAGTTGACCAC-3’

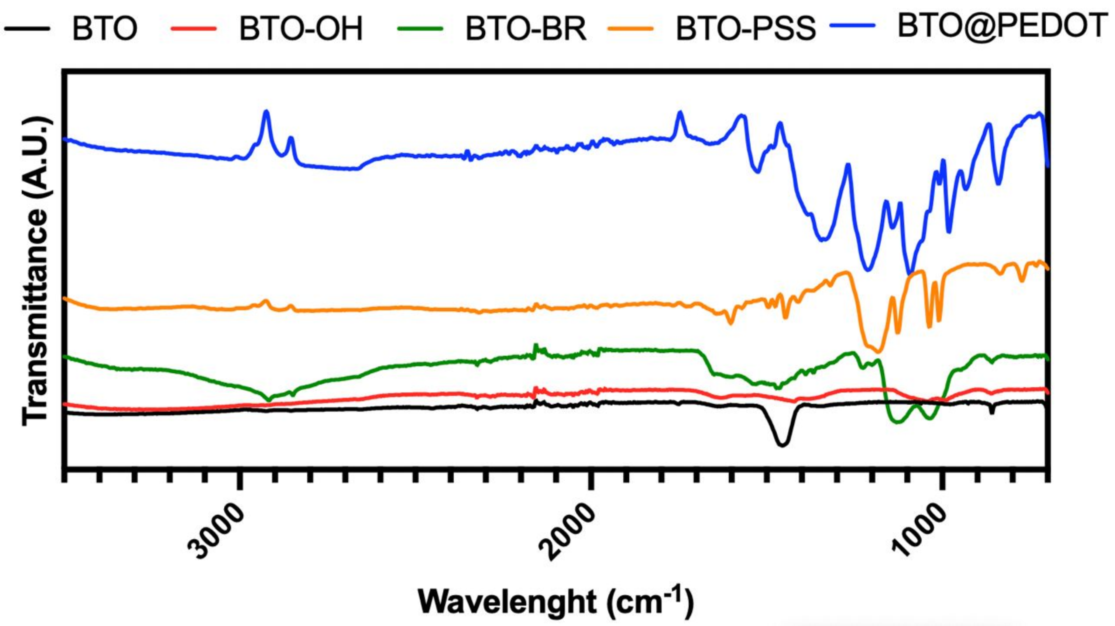

Figure S1. FTIR spectra of naked BTO, BTO-OH, BTO-Br and BTO-PSS intermediaries and BTO@PEDOT NPs.

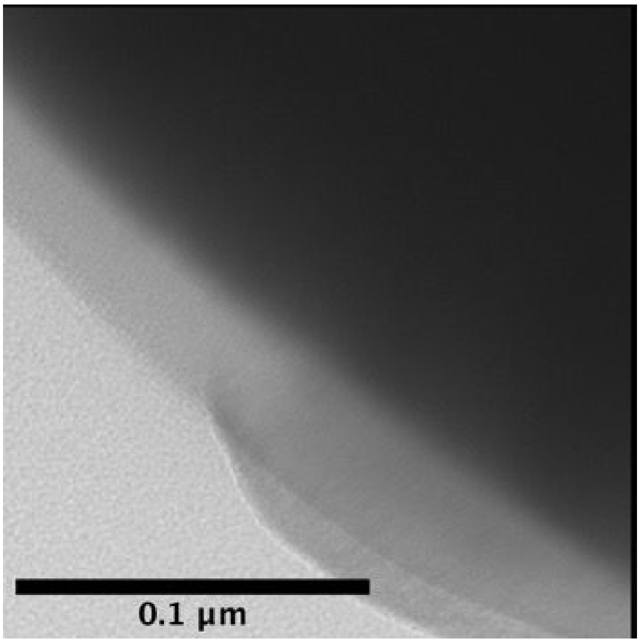

Figure S2. TEM image of the particles after BTO-PSS modification

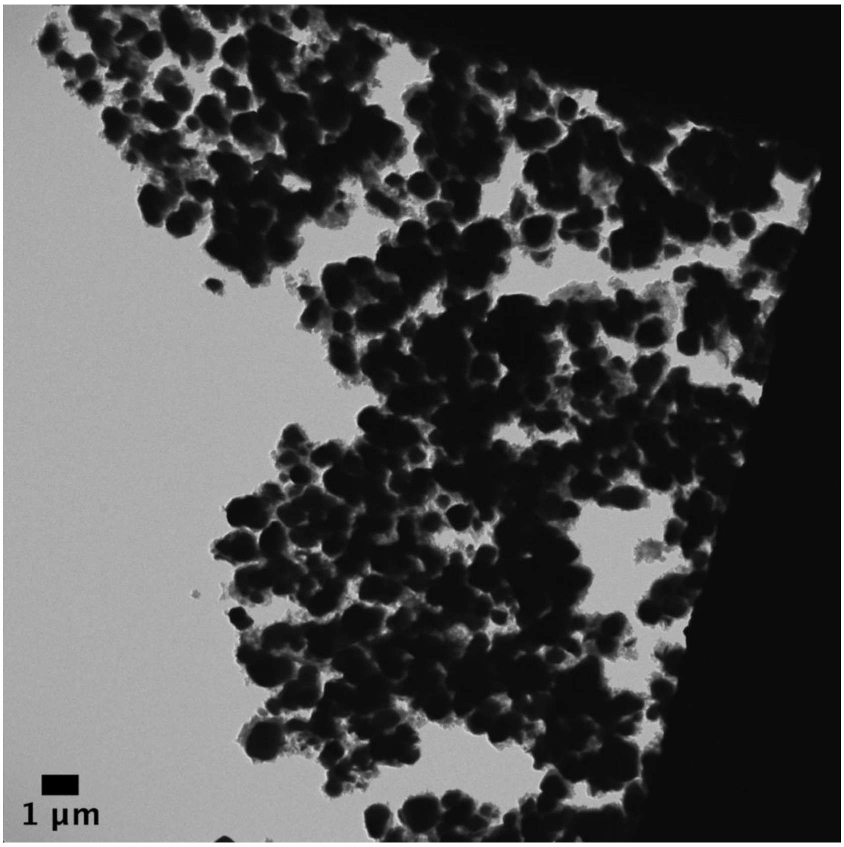

Figure S3. TEM image of the 500 nm BTO@PEDOT NPs.

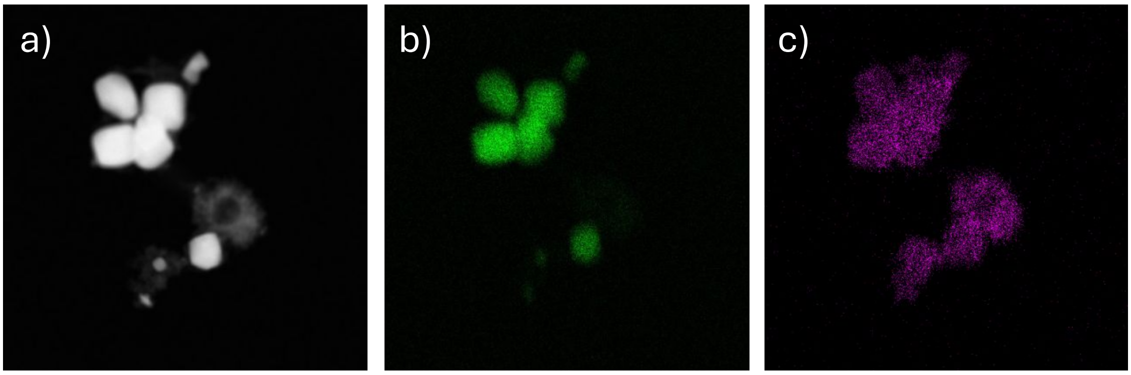

Figure S4. EDX of the (a) 500 nm BTO after BTO-PEDOT modification showing the (b) Cu and (c) Fe distribution

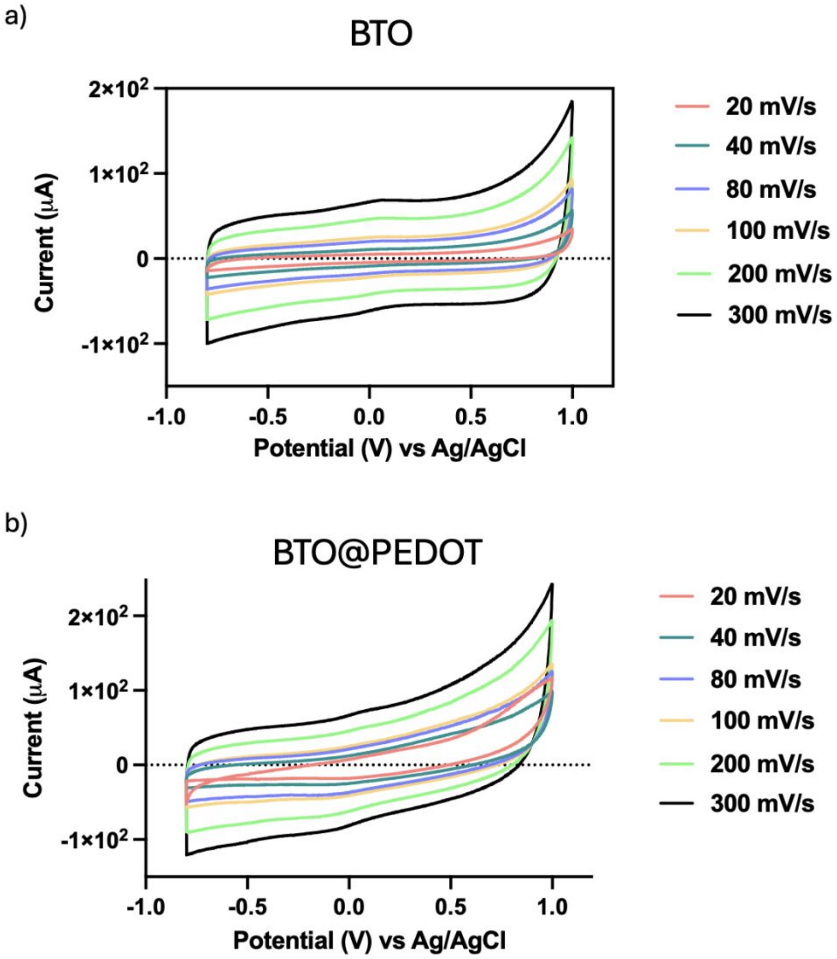

Figure S5. Cyclic voltammetry (CV) curves of a) BTO and b) BTO@PEDOT NPs at different sweep scan rates.

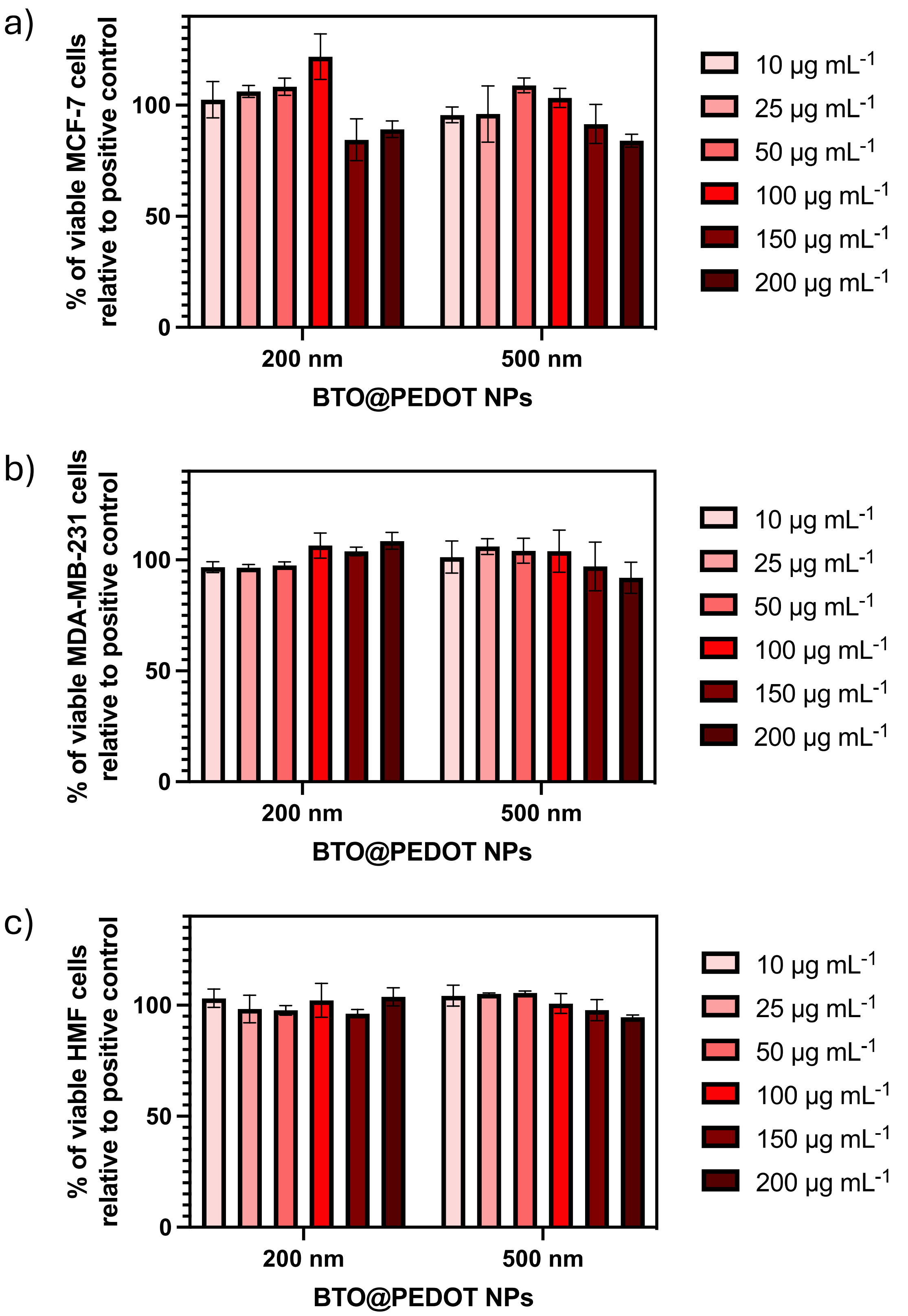

Figure S6. WST-8 metabolic activity assay of a) MCF-7, b) MDA-MB-231 and c) HMF cells incubated with increasing concentrations of 200 nm and 500 nm BTO@PEDOT NPs. Positive control consisted on cells incubated in cell culture media and assumed as 100% viability. Data are shown as mean ± SD (n=3)

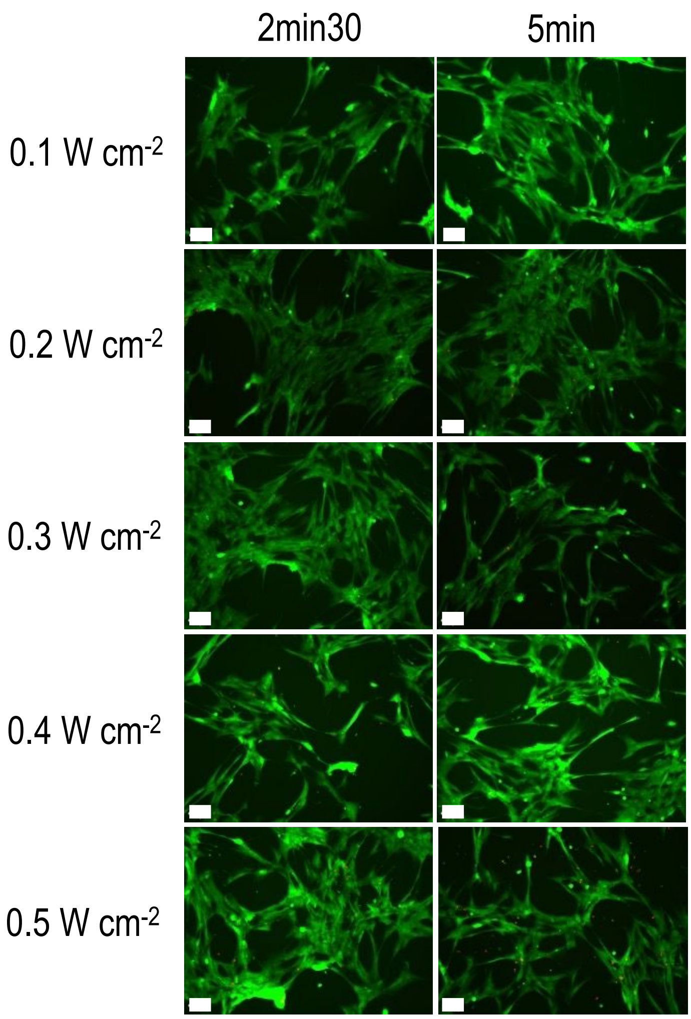

Figure S7. Representative fluorescence microscopy images of the HMF cells under different parameters of ultrasound (US) stimulation after live/dead staining. Scale bar 100 µm.

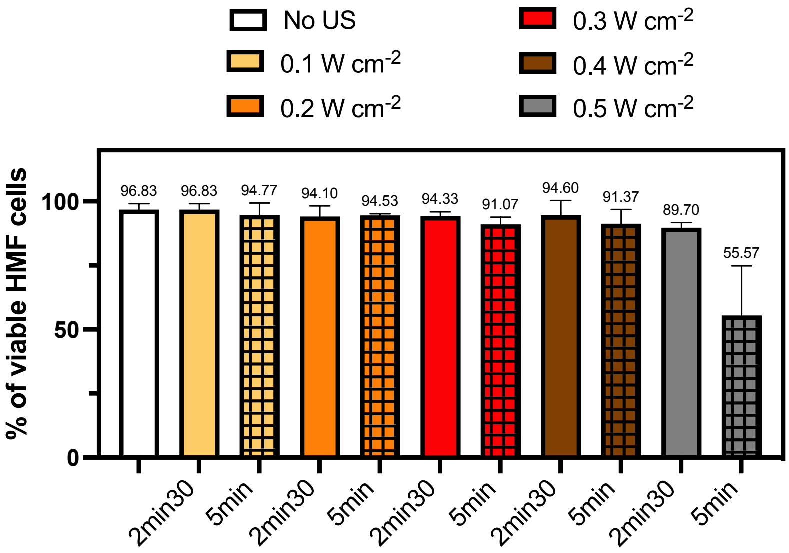

Figure S8. Calculation of the percentage of viable HMF cells using different US stimulation parameters. Data are shown as mean ± SD (n=3)

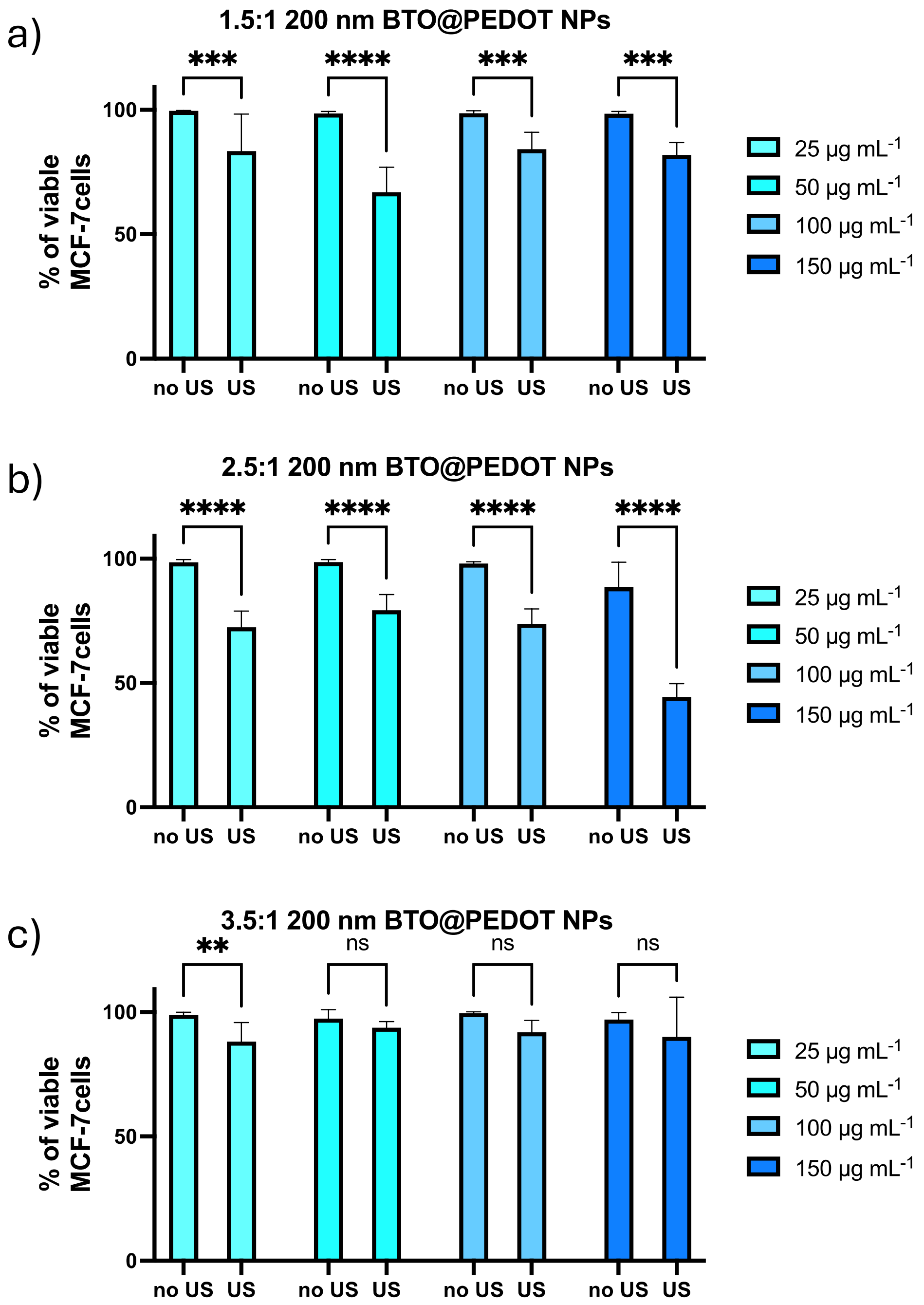

Figure S9. Percentage of viable MCF-7 cells at increasing concentration of 200 nm BTO@PEDOT NPs with and without US stimulation using a) 1.5:1, b) 2.5:1 and c) 3.5:1 PEDOT:PSS ratios. Statistical analysis was performed using one-way ANOVA. Data are shown as mean ± SD (N=3, n=2)

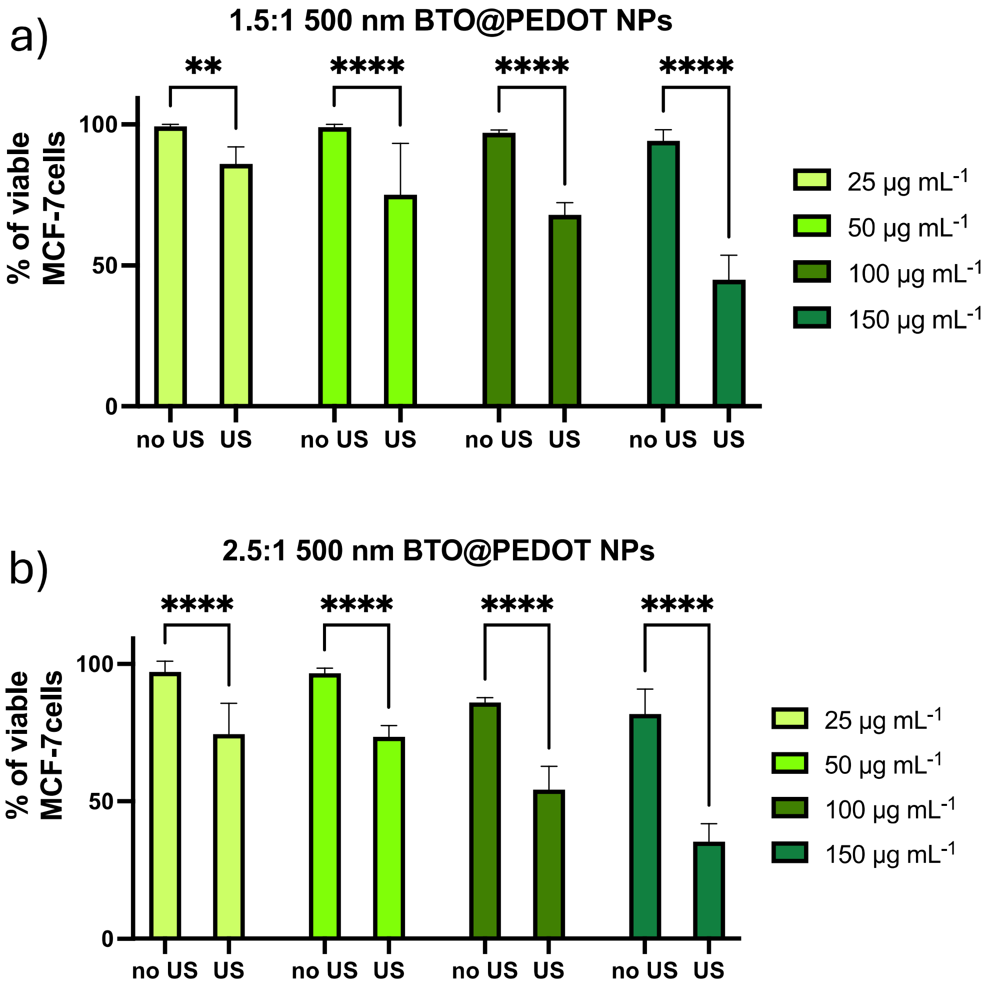

Figure S10. Percentage of viable MCF-7 cells at increasing concentration of 500 nm BTO@PEDOT NPs with and without stimulation using a) 1.5:1 and b) 2.5:1 EDOT:SSNa ratios. Statistical analysis was performed using one-way ANOVA. Data are shown as mean ± SD (N=3, n=2)

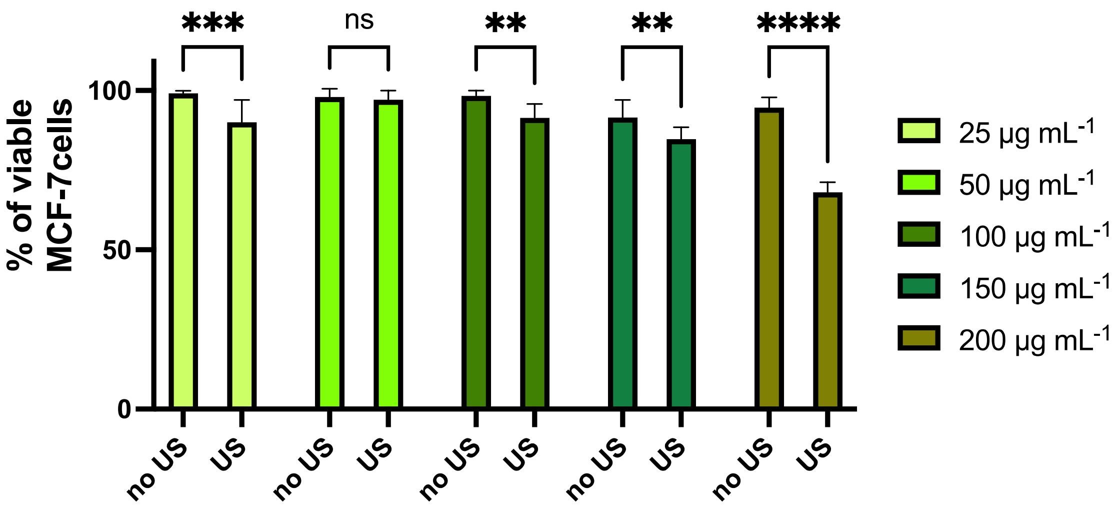

Figure S11. Percentage of viable MCF-7 cells at increasing concentration of 500 nm BTNPs with and without US stimulation. Statistical analysis was performed using one-way ANOVA. Data are shown as mean ± SD (N=3, n=2)

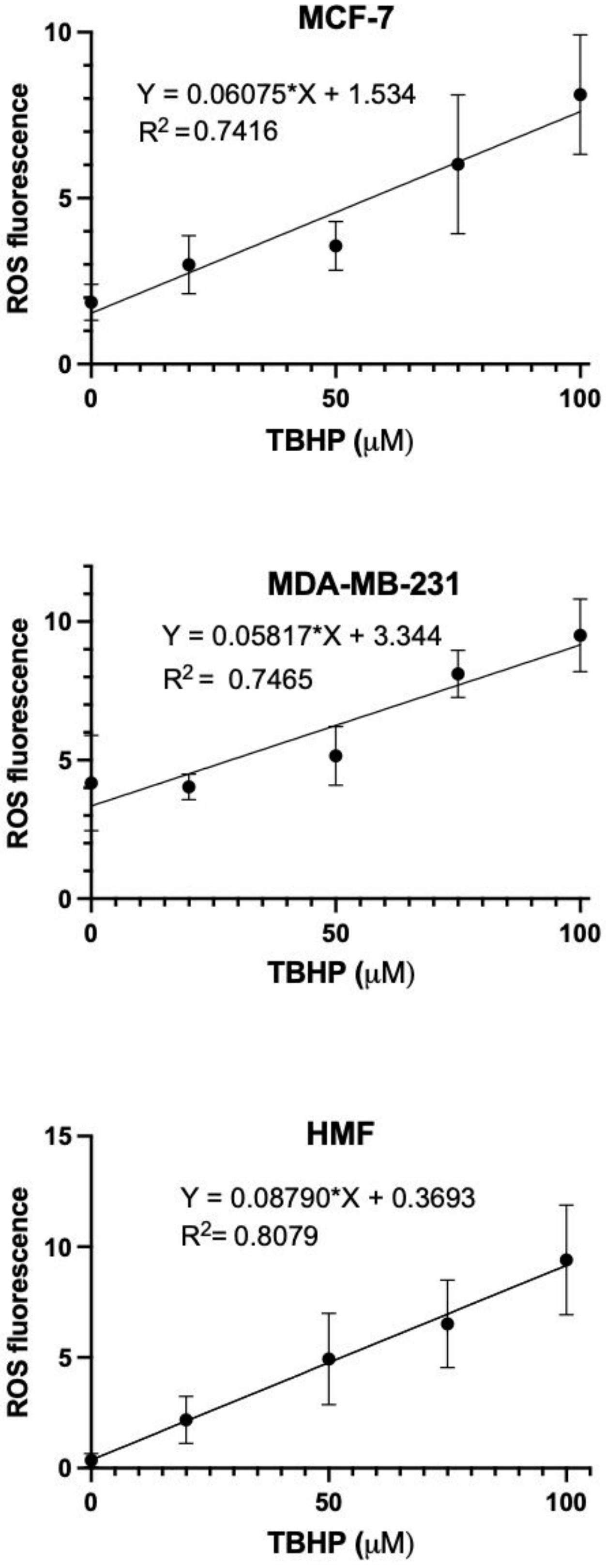

Figure S12. Calibration curve of ROS at increasing concentrations of TBHP for MCF-7, MDA-MB-231 and HMF.

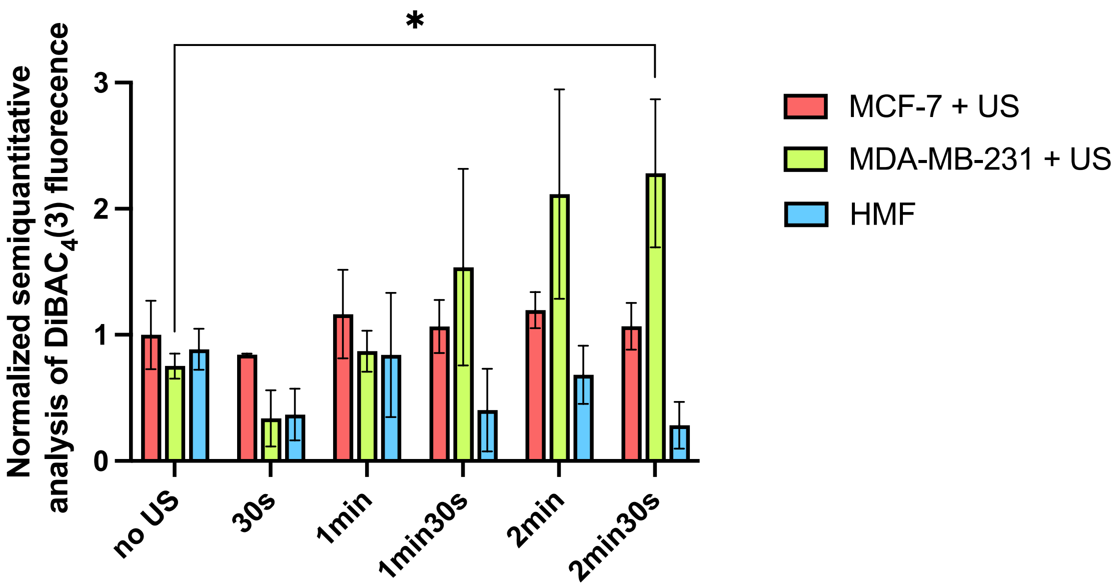

Figure S13. DiBAC$_4$(3) fluorescence data of MCF-7, MDA-MB-231, and HMF under US stimulation without BTO@PEDOT NPs.

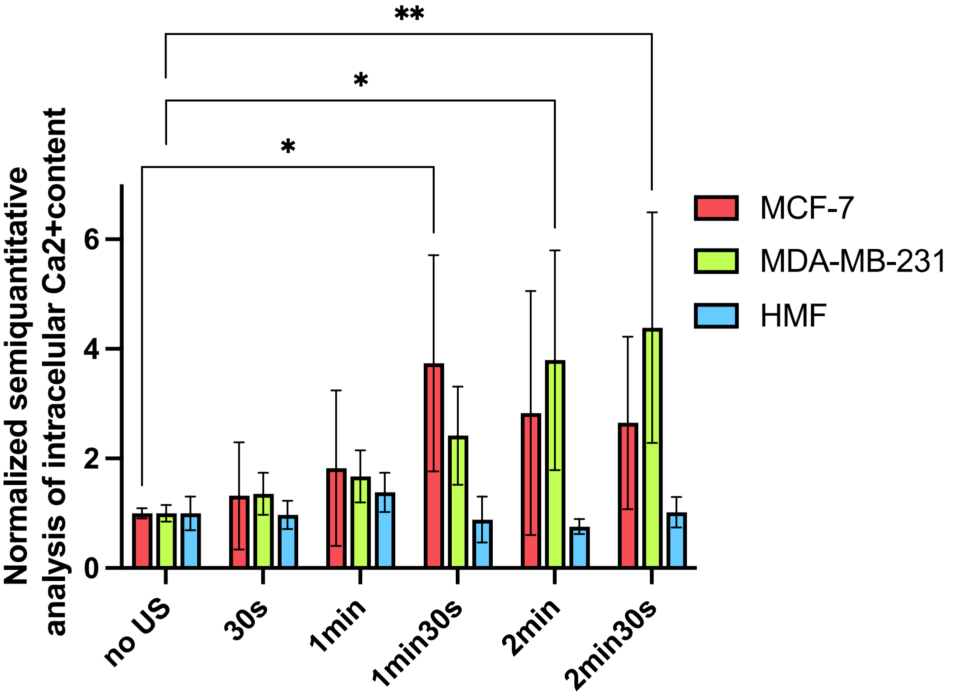

Figure S14. Intracellular calcium fluorescence data of MCF-7, MDA-MB-231, and HMF under US stimulation without BTO@PEDOT NPs.

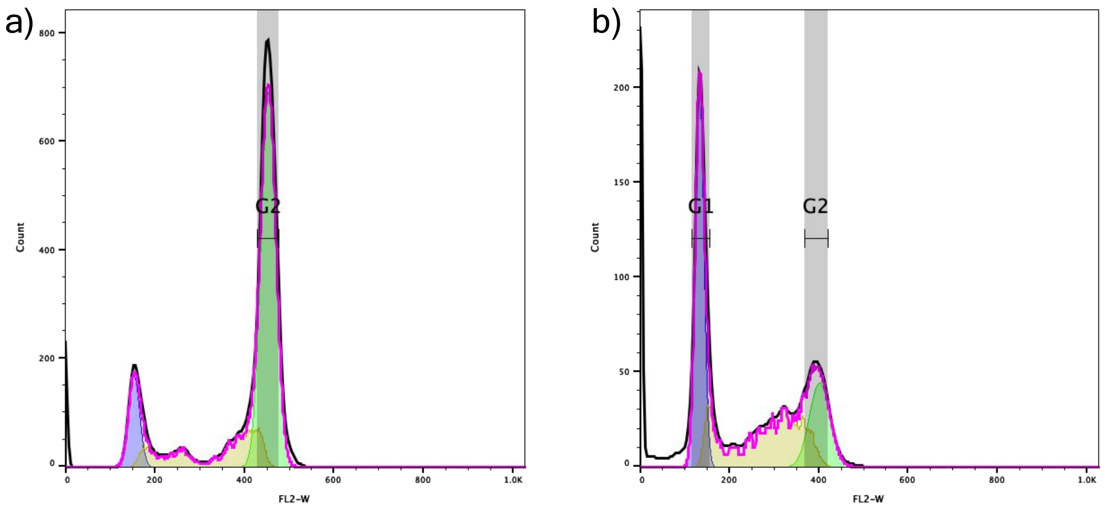

Figure S15. Evaluation of the cell cycle of MCF-7 cells performed by flow cytometry of (a) control cells cultivated in culture media and (b) cells after US stimulation in the presence of BTO@PEDOT NPs

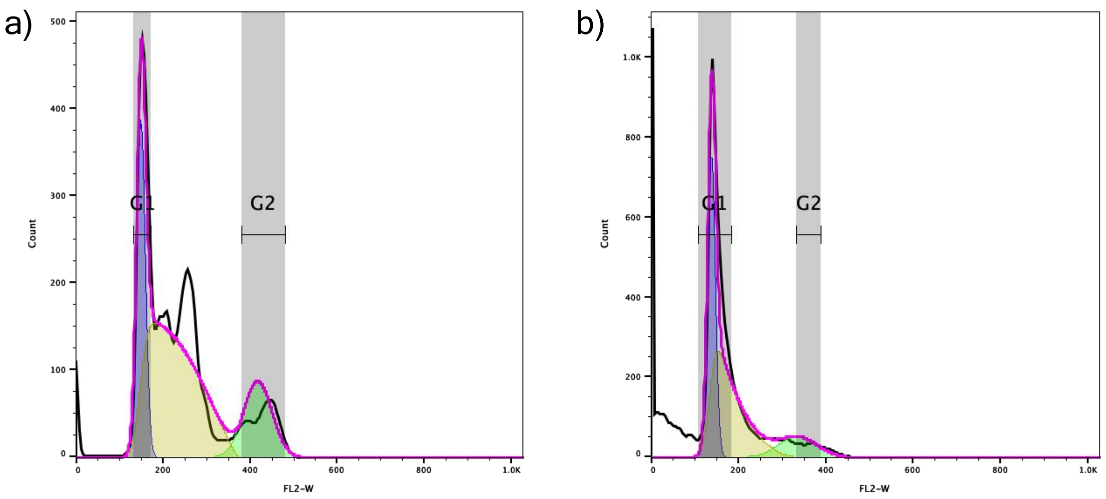

Figure S16. Evaluation of the cell cycle of MDA-MB-231 cells performed by flow cytometry of (a) control cells cultivated in culture media and (b) cells after US stimulation in the presence of BTO@PEDOT NPs

a) b)

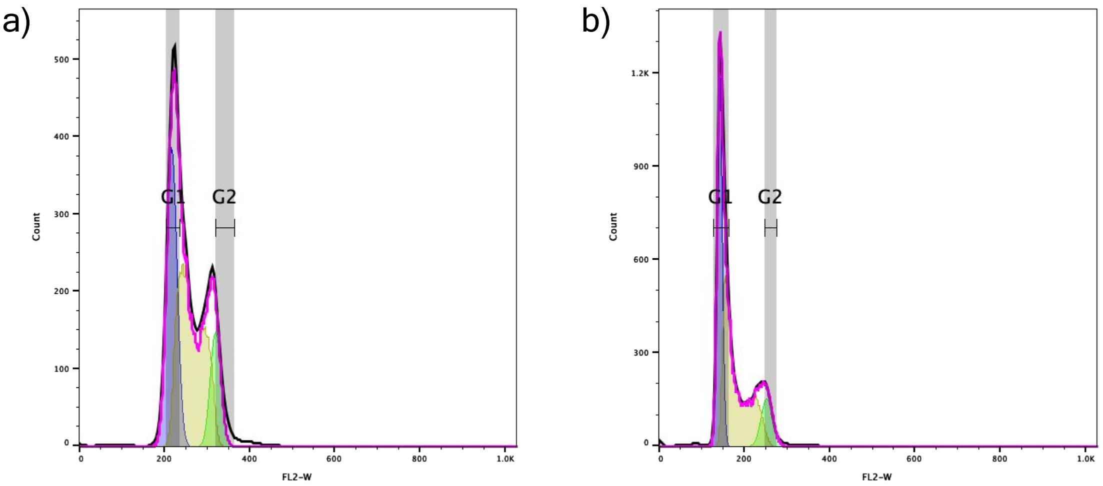

Figure S17. Evaluation of the cell cycle of HMF cells performed by flow cytometry of (a) control cells cultivated in culture media and (b) cells after US stimulation in the presence of BTO@PEDOT NPs
